# Distinct anti-NP, anti-RBD and anti-Spike antibody profiles discriminate death from survival in COVID-19

**DOI:** 10.3389/fimmu.2023.1206979

**Published:** 2023-10-09

**Authors:** Carolina do Prado Servian, Mônica Spadafora-Ferreira, Déborah Carolina Carvalho dos Anjos, Adriana Oliveira Guilarde, Antonio Roberto Gomes-Junior, Moara Alves Santa Bárbara Borges, Letícia Carrijo Masson, João Marcos Maia Silva, Matheus Henrique Assis de Lima, Brenda Grazielli Nogueira Moraes, Sueli Meira Souza, Luiz Eterno Xavier, Denise Cristina André de Oliveira, João Victor Batalha-Carvalho, Ana Maria Moro, Anamélia Lorenzetti Bocca, Irmtraut Araci Hoffmann Pfrimer, Nádia Lago Costa, Valéria Christina de Rezende Feres, Fabiola Souza Fiaccadori, Menira Souza, Luiz Gustavo Gardinassi, Edison Luiz Durigon, Pedro Roosevelt Torres Romão, Soraia Attie Calil Jorge, Verônica Coelho, Viviane Fongaro Botosso, Simone Gonçalves Fonseca

**Affiliations:** ^1^ Departamento de Biociências e Tecnologia, Instituto de Patologia Tropical e Saúde Pública, Universidade Federal de Goiás, Goiânia, GO, Brazil; ^2^ Laboratório de Imunogenética, Instituto Butantan, São Paulo, SP, Brazil; ^3^ Departamento de Patologia Tropical e Dermatologia, Instituto de Patologia Tropical e Saúde Pública, Universidade Federal de Goiás, Goiânia, GO, Brazil; ^4^ Hospital das Clínicas, Faculdade de Medicina, Universidade Federal de Goiás, Goiânia, GO, Brazil; ^5^ Vigilância Epidemiológica da Secretaria Municipal de Saúde de Goiânia, Goiânia, GO, Brazil; ^6^ Laboratório Prof^a^ Margarida Dobler Komma, Instituto de Patologia Tropical e Saúde Pública, Universidade Federal de Goiás, Goiânia, GO, Brazil; ^7^ Laboratório de Virologia, Instituto Butantan, São Paulo, SP, Brazil; ^8^ Laboratório de Biofármacos, Instituto Butantan, São Paulo, SP, Brazil; ^9^ Instituto de Investigação em Imunologia – Instituto Nacional de Ciências e Tecnologia (III-INCT), São Paulo, SP, Brazil; ^10^ Departamento de Biologia Celular, Instituto de Biologia, Universidade de Brasília, Brasília, DF, Brazil; ^11^ Escola de Ciências Médicas e da Vida, Pontifícia Universidade Católica de Goiás, Goiânia, GO, Brazil; ^12^ Faculdade de Odontologia, Universidade Federal de Goiás, Goiânia, GO, Brazil; ^13^ Faculdade de Farmácia, Universidade Federal de Goiás, Goiânia, GO, Brazil; ^14^ Departamento de Microbiologia, Instituto de Ciências Biomédicas, Universidade de São Paulo, São Paulo, SP, Brazil; ^15^ Laboratório de Imunologia Celular e Molecular, Programa de Pós-Graduação em Ciências da Saúde, Programa de Pós-Graduação em Biociências, Universidade Federal de Ciências da Saúde de Porto Alegre, Porto Alegre, RS, Brazil; ^16^ Laboratório de Biotecnologia Viral, Instituto Butantan, São Paulo, SP, Brazil; ^17^ Laboratório de Imunologia, Instituto do Coração (InCor), Universidade de São Paulo, Faculdade de Medicina, São Paulo, SP, Brazil; ^18^ Laboratório de Histocompatibilidade e Imunidade Celular, Hospital das Clínicas Hospital da Clínicas, Faculdade de Medicina, Universidade de São Paulo (HCFMUSP), São Paulo, SP, Brazil

**Keywords:** COVID-19, SARS-CoV-2, antibodies, receptor binding domain (RBD), spike protein (S), nucleocapsid protein (N), clinical severity

## Abstract

**Introduction:**

Infection by severe acute respiratory syndrome coronavirus 2 (SARS-CoV-2) induces rapid production of IgM, IgA, and IgG antibodies directed to multiple viral antigens that may have impact diverse clinical outcomes.

**Methods:**

We evaluated IgM, IgA, and IgG antibodies directed to the nucleocapsid (NP), IgA and IgG to the Spike protein and to the receptor-binding domain (RBD), and the presence of neutralizing antibodies (nAb), in a cohort of unvaccinated SARS-CoV-2 infected individuals, in the first 30 days of post-symptom onset (PSO) (T1).

**Results:**

This study included 193 coronavirus disease 2019 (COVID-19) participants classified as mild, moderate, severe, critical, and fatal and 27 uninfected controls. In T1, we identified differential antibody profiles associated with distinct clinical presentation. The mild group presented lower levels of anti-NP IgG, and IgA (vs moderate and severe), anti-NP IgM (vs severe, critical and fatal), anti-Spike IgA (vs severe and fatal), and anti-RBD IgG (vs severe). The moderate group presented higher levels of anti-RBD IgA, comparing with severe group. The severe group presented higher levels of anti-NP IgA (vs mild and fatal) and anti-RBD IgG (vs mild and moderate). The fatal group presented higher levels of anti-NP IgM and anti-Spike IgA (vs mild), but lower levels of anti-NP IgA (vs severe). The levels of nAb was lower just in mild group compared to severe, critical, and fatal groups, moreover, no difference was observed among the more severe groups. In addition, we studied 82 convalescent individuals, between 31 days to 6 months (T2) or more than 6 months (T3), PSO, those: 12 mild, 26 moderate, and 46 severe plus critical. The longitudinal analyzes, for the severe plus critical group showed lower levels of anti-NP IgG, IgA and IgM, anti-Spike IgA in relation T3. The follow-up in the fatal group, reveals that the levels of anti-spike IgG increased, while anti-NP IgM levels was decreased along the time in severe/critical and fatal as well as anti-NP IgG and IgA in several/critical groups.

**Discussion:**

In summary, the anti-NP IgA and IgG lower levels and the higher levels of anti-RBD and anti-Spike IgA in fatal compared to survival group of individuals admitted to the intensive care unit (ICU). Collectively, our data discriminate death from survival, suggesting that anti-RBD IgA and anti-Spike IgA may play some deleterious effect, in contrast with the potentially protective effect of anti-NP IgA and IgG in the survival group.

## Introduction

The new coronavirus SARS-CoV-2, the etiological agent of COVID-19, is one of the main pathogens that especially targets the human respiratory system (1). COVID-19 has become a public health problem due to high rates of morbidity and mortality, causing millions of deaths and a long-term health burden (2).

The SARS-CoV-2 particle has four structural proteins: spike (S), envelope (E), membrane glycoprotein (M), and nucleocapsid protein (N). To exert its pathogenic mechanism, SARS-CoV-2 binds to host cells through a trimeric glycoprotein that recognizes the angiotensin converting enzyme 2 (ACE2), the S protein, which is cleaved into two domains S1 and S2 (3). The S1 domain contains the receptor binding domain (RBD), which is essential for viral binding to receptor human ACE2 (hACE2) and the establishment of cellular infection (4–6), considered a target for neutralizing antibodies (nAbs). Antibodies that bind to the spike protein, specifically to the RBD and N-terminal domains, inhibit the binding of viruses to cells by neutralizing viral particles (7).

Different profiles of anti-SARS-CoV-2 production and antibody levels and dynamics have been associated with distinct mild or severe clinical outcomes over time (8, 9). However, the underlying mechanisms contributing to better or worse outcomes are still being studied.

Generally, in the early stages of SARS-CoV-2 infection, IgM is the main antibody, IgA- and IgG-mediated protection prevents pathogens from binding and invading the host cells, and IgG is the antibody that has a longer duration in the blood (10, 11). It has been suggested that high levels of IgM and IgG antibodies against the S1 and N proteins, in the first 15 days post-symptom onset (PSO), is considered a risk factor for a more severe clinical outcomes, since these antibodies were detected at higher levels in COVID-19 patients admitted to the intensive care unit (ICU) and in those who died (12–14). High titers of anti-Spike IgM have been reported around 10 to 12 days after symptoms began, with a significant reduction after the 18^th^ day (15). Anti-spike IgA antibodies show induction in the first week of infection and peaking levels around day 20, concomitantly with an increased number of IgA-anti-SARS-CoV-2 secreting plasmablasts (16). There are still several contradictory and unknown issues regarding the levels of anti-SARS-Cov-2 antibodies and the severity of clinical outcomes.

Neutralizing antibodies have mostly been observed to persist up to 180 days after the onset of symptoms (17) and play a critical role in blocking viral entrance into cells. The neutralizing capacity of anti-SARS-CoV-2 antibodies has been reported to be predominantly mediated by IgA, early in infection, and they are seven times more effective than IgG (16). Also, IgM, IgG1 and IgA1 showed neutralizing activity against Spike and RBD proteins early after infection (18). Longitudinal analysis of antibody dynamics in COVID-19 convalescents revealed neutralizing responses up to 16 months after infection (19). In addition, anti-RBD and anti-spike IgG antibodies in hospitalized COVID-19 patients have also been shown to display important participation in complement deposition but a lower capacity in phagocytosis promotion, in comparison to non-hospitalized individuals (20).

In this work, we investigated the IgM, IgA and IgG antibody profiles directed to SARS-CoV-2 antigens, as well as antibody neutralizing capacity, in COVID-19 individuals with different disease outcomes, aiming to determine whether specific profiles were associated with COVID-19 severity or recovery, suggesting potential beneficial versus deleterious antibody functions in COVID-19. We found that higher levels of anti-RBD and anti-Spike IgA distinguished fatal from survival in individuals admitted to the ICU, suggesting that these antibodies may play some deleterious effect in the long run, in contrast with the potentially protective effect of anti-NP IgA and IgG that were higher in survival individuals.

## Materials and methods

### Ethics approval and consent to participate

Participants, family members, or legal guardians have provided oral recorded informed consent, in accordance with the regulations of the Human Ethical Committee from Hospital das Clínicas, Faculdade de Medicina of Universidade Federal de Goiás (UFG), Goiânia, Goiás, Brazil, and the research protocol was approved by Ethical Appreciation (CAAE: 30804220.2.0000.5078). The sample size was determined by the convenience of sampling, availability at partner hospitals, agreement to participate, and pandemic conditions within the local community.

### Study cohort

Sample collection was conducted from June 2020 to June 2021. COVID-19 positive individuals (n = 193), with positive diagnosis of SARS-CoV-2 infection by real-time reverse-transcriptase quantitative polymerase chain reaction (RT-qPCR) from nasopharyngeal swabs and/or rapid test assays to detect IgM/IgG (Eco Diagnostics) were enrolled in the study, all before the initial vaccination. Blood samples were obtained in the first 30 days PSO for all positive individuals and a follow-up was conducted in a subset of individuals (n=82) with sample collection up to T1: up to 30 days PSO, T2: 1–6 months PSO, and T3: more than 6 months PSO. The samples were collected at COVID-19 wards and the Intensive Care Unit (ICU) at Hospital das Clínicas, Universidade Federal de Goiás, Goiânia. Brazil. For individuals who were not hospitalized and recovered, blood samples were collected at Laboratório Prof^a^ Margarida Dobler Komma, Instituto de Patologia Tropical e Saúde Pública, Universidade Federal de Goiás, Goiânia, Brazil. Additionally, 27 individuals, negative for SARS-CoV-2 by RT-qPCR in nasopharyngeal swabs and pre-vaccination, were enrolled as controls. SARS-CoV-2 positive participants were categorized according to National Institute of Health (NIH), USA, classification for COVID-19 (21, 22) as: mild disease (individuals who had any of the various signs/symptoms but did not have shortness of breath, dyspnea, or abnormal chest imaging, can be managed in an ambulatory or at home), moderate disease (radiologically confirmed pneumonitis, hospitalization and oxygen therapy), severe disease (dyspnea, respiratory frequency ≥30 breaths/min, oxygen saturation [SpO2] ≤93%, and/or lung infiltrates >50% within 24 - 48 hours), and critical disease (treatment in ICU, or complications by other organ failure and/or mechanical ventilation). The fatal group included all participants who required ICU and died. Peripheral blood samples from all participants were collected, and serum was separated and stored at −80°C.

### SARS-CoV-2 RNA extraction and RT-qPCR

Ribonucleic acid (RNA) extraction was performed using the commercial QIAamp® Viral RNA Mini Kit (Qiagen, Germany), following the manufacturer’s protocol. After RNA extraction, samples were submitted to real-time polymerase chain reaction post reverse transcription (RT-qPCR) using the Promega GoTaq® Probe 1-Step RT-qPCR System, according to the manufacturer’s protocol (23). Primers and probes targeted two regions of the N gene (N1 and N2) from SARS-CoV-2 and the human RNAse P (RP) gene, and IDT (Integrated DNA Technologies, Iowa, USA). All samples that presented a cycle threshold (Ct) lower than 40 (for N1, N2, and RP targets) were positive for SARS-CoV-2 RNA. Viral loads in genomic copies (GC) per mL/g of clinical specimens were estimated based on a standard curve of serial dilutions (10^6^ to 10^0^ GC/µL) of the synthetic positive control nCoVPC (severe acute respiratory syndrome coronavirus 2 isolate Wuhan-Hu-1, complete genome, GenBank: NC_045512.2) from Integrated DNA Technologies (24).

### Neutralizing antibody assay

A cytopathic effect-based virus neutralization test (CPE-VNT) was performed using 96-well plates, as previously described by Botosso (25). Briefly, serum samples were initially inactivated for 30 minutes at 56°C and subsequently diluted in DMEM containing 2.5% fetal bovine serum from 1/20 to 1/2560. The sera were then mixed vol/vol with 100 tissue culture infectious doses, 50% endpoint (TCID50) of the virus (SARS-CoV-2 wild-type variant B.1.1.28 - MT126808) and pre-incubated at 37° C for 1 h for neutralization. The serum/virus mixture was transferred onto the confluent VERO ATCC CCL-81.4 cell monolayer and incubated for 3 days at 37°C with 5% CO2. After incubation, the plates were analyzed by light microscopy for the presence or absence of SARS-CoV-2 CPE. For confirmation, plates were fixed and stained with amido black (0.1% amido black solution [w/w] with 5.4% acetic acid, 0.7% sodium acetate) for 30 minutes and analyzed to determine the titer. nAb titer (VTN100) is considered the highest serum dilution neutralizing virus growth. Internal positive and negative controls were added to each run. All CPE-VNT procedures were performed in a Biosafety Level 3 at the Institute of Biomedical Science, University of São Paulo, laboratory following the World Health Organization recommendations. nAb titers were transformed in logarithm (log) for normalization.

### ELISA to detect SARS-CoV-2 antibodies

Enzyme-linked immunosorbent assay (ELISA) was performed using 96-well high-binding polystyrene COSTAR microplates (Corning, NY, USA, #3590) coated overnight at 4°C with 2.0 µg/mL recombinant Spike protein (26), 1.0 µg/mL NP (27) or 1.5 µg/mL RBD expressed according to Amanat et al. (9) diluted in 0.1 M sodium carbonate-sodium bicarbonate buffer, pH 9.6. Briefly, unbound proteins were removed, followed by blocking with 1% albumin bovine serum (BSA, Sigma) and 5% nonfat dry milk in phosphate buffered saline containing 0.02% Tween 20 (PBST) for 2 or 3 hours at 37°C. After washing three times with PBST, plates were incubated for 45 min at 37°C with 50 μL heat inactivated serum samples (56°C for 30 minutes) diluted to 1:50 for IgA and 1:100 for IgG, in PBST with 0.25% BSA and 5% nonfat dry milk. Each sample was assayed in duplicate. After another series of washing, the plates were incubated for 30 min at 37°C with peroxidase-conjugated goat anti-human IgA (Sigma A0295, 1:2500), IgG (Sigma A0170, 1:4000), or IgM (Sigma A6907, 1:3000) secondary antibodies. After washing, 50 μL of 3,3’,5,5’-Tetramethylbenzidine (Life Technologies, Cat. no. 002023) were added to each well and incubated for 10 minutes at room temperature. The reaction was stopped by adding 25μL of 2 N of sulfuric acid. Optical density (OD) was measured at 450 nm using a microplate reader (Labsystems Multiskan, Thermo Scientific, USA). Values were determined as OD minus blank, and the cutoff (CO) was determined as the average OD of samples pre-pandemics or negative ± 2× standard deviation. Each plate we included positive serum for control obtained by SARS-CoV-2 confirmed by RT-PCR. The results were normalized across experiments and transformed as the ratio of sample/cutoff (S/CO). The frequency distribution of antibody detection was calculated as positive when S/CO was higher than or equal to 1.2, and negative detection when S/CO was less than 1.2 (28).

### Statistical analysis

All analyses were conducted using GraphPad Prism version 9 for Windows (GraphPad Software, La Jolla California USA). Receiver-operating characteristic (ROC) analyses were performed using MetaboAnalyst. For the correlograms, based on the Spearman correlation, were generated with the package *corrplot* for R studio software (version)”. The distribution patterns of the variables were checked using the Kolmogorov–Smirnov and Shapiro–Wilk tests. For frequency calculations, we used the Fisher exact test. For comparisons between paired groups, we used the non-parametric Wilcoxon Matched–Pairs signed-rank test. Unpaired groups were analyzed using the non-parametric Mann–Whitney U test. Multiple group comparisons were analyzed by running a non-parametric Kruskal–Wallis statistical test and were corrected using Dunn’s and Dunnett’s methods. Spearman correlation coefficients and nonlinear regression analysis were used to assess significance. For all tests, a p value < 0.05 was considered significant.

## Results

### Clinical and demographic characteristics of COVID-19 individuals and controls

To determine the profile of the SARS-CoV-2 specific humoral immune response, we recruited 27 healthy individuals (control group: negative for SARS-CoV-2 RT-qPCR from nasopharyngeal swabs) and 193 individuals with COVID-19 between June 2020 and February 2021. Part of the samples of cohort was sequenced and the predominant circulating strain was classified as B.1.1.33 lineage of SARS-CoV-2 in that period, as described in our other work (23). All participants were unvaccinated to COVID-19. For our initial analysis, the cohort was first stratified based on disease severity. SARS-CoV-2 positive participants were categorized as: mild (n = 37, from these 26 were not hospitalized participants), moderate (n = 43), severe (n = 63), critical (n = 14), death (n = 36) and recovered (n = 84), according to the NIH classification for COVID-19 (21, 22). It is worth to mention that some patients with mild disease were hospitalized due to decompensation of the underlying disease.

Among all COVID-19 participants, the median age was 58 years (interquartile range [IQR] = 45-71) and was not different from the control group (median=56 years, IQR=50-60). In the COVID-19 group, the median age for mild disease was 33 years, 55 years for moderate disease, 58 years for severe disease, 48 years for critical disease, and 63 years for fatal individuals (**Table 1**).

**Table 1:** Associations between clinical and demographic data of participants and hospital care and interventions (n=220).

Baseline Variable	Control N=27	All patients N=193	Mild N=37	Moderate N=43	Severe N=63	Critical N=14	Fatal N=36	*p* value
Demographic characteristics								
**Age, median (IQR)**	56 (50-60)	58 (45-71)	33 (25-46)	55 (44-74)	58 (46-71)	48 (34-63)	63 (56-71)	^a,f,g,i^
Sex, no. (%)								
**Male**	9 (33)	95 (49)	12 (32)	22 (51)	36 (57)	4 (29)	21 (58)	^g,i^
**Female**	18 (67)	98 (51)	25 (68)	21 (49)	27 (43)	10 (71)	15 (42)	
**Days post symptom onset collection, median (IQR)**	–	14 (10-18)	9 (6-11)	14 (11-18)	14 (12-18)	15 (10-23)	17 (15-20)	^f,g,i,h^
Comorbidities and risk factors, no. (%)								
**Hypertension**	7 (29.2)	64 (33.2)	5 (13.5)	15 (34.9)	24 (38.1)	5 (35.7)	15 (42.0)	^f,g,i^
**Diabetes mellitus**	1 (3.7)	46 (23.8)	3 (8.1)	13 (30.2)	16 (25.4)	4 (29.5)	10 (28.0)	^b,c,d,e,f,g,i^
**Obesity**	6 (22.2)	31 (16.1)	–	7 (16.3)	15 (23.8)	4 (29.5)	5 (14.0)	ns
**Heart disease**	3 (11.1)	18 (9.3)	2 (5.4)	10 (23.3)	16 (25.4)	-	7 (19.4)	^f,g^
**Pregnancy**	–	28 (28.6)	9 (36.0)	6 (29.0)	7 (23.0)	2 (2.0)	4 (27.0)	ns
Symptoms, no. (%)								
**Cough**	**-**	102 (52.8)	15 (40.5)	27 (63.0)	43 (68.3)	8 (57.1)	24 (66.7)	^g,i^
**Dyspnea**	**-**	91 (47.1)	12 (32.4)	19 (44.2)	43 (68.3)	8 (57.1)	27 (75.0)	^g,i,j,l^
**Fever**	**-**	81 (42.0)	13 (35.1)	23 (53.5)	29 (46.0)	8 (57.1)	28 (77.8)	^i,l,n^
**Myalgia/Arthralgia**	**-**	57 (29.5)	11 (29.7)	13 (30.2)	28 (44.4)	2 (14.3)	12 (33.3)	^ns^
**Headache**	**-**	49 (25.4)	11 (29.7)	14 (32.5)	19 (30.2)	2 (14.3)	11 (30.6)	^ns^
**Asthenia**	**-**	45 (23.3)	8 (21.6)	9 (20.9)	19 (30.2)	4 (28.5)	14 (38.9)	^ns^
**Diarrhea**	**-**	29 (15.0)	8 (21.6)	2 (4.6)	17 (27.0)	–	3 (8.3)	^f,j,n^
**Anosmia**	**-**	18 (9.3)	6 (16.2)	6 (14.0)	4 (6.4)	1 (7.1)	1 (2.8)	^ns^
**Chest pain**	**-**	15 (7.8)	2 (5.4)	1 (2.3)	9 (14.3)	–	4 (11.1)	^j^
Hospital support, no. (%)								
**Infirmary**	**-**	94 (48.7)	11 (29.7)	43 (100)	40 (63.5)	–	**-**	^f, j^
**ICU**	**-**	73 (37.8)	-	-	23 (36.5)	14 (100.0)	36 (100.0)	^m,n^
Respiratory support received, no. (%)								
**Nasal Catheter**	**-**	100 (51.8)	11 (29.7)	42 (97.7)	47 (74.6)	-	-	^j^
**Non-rebreathing mask**	**-**	22 (11.4)	–	1 (2.3)	16 (25.4)	–	5 (14.0)	^j^
**Invasive mechanical ventilation**	**-**	45 (23.3)	-	-		14 (100.0)	31 (86.0)	ns

no., number, or values; ICU, Intensive Care Unit; s, second; IQR, Interquartile range; ns, not significant. Comparison of the control (healthy participants) with all patients. Categorical variables represented as number (percentage) and compared using Fisher’s exact test. Continuous variables represented as median (interquartile range) and compared using one-way analysis of variance (ANOVA) and Kruskal-Wallis test. *p<0.05. Significance comparing control versus mild = a, control versus moderate = b, control versus severe = c, control versus critical = d, control versus fatal = e; mild versus moderate = f, mild versus severe = g, mild versus critical = h, mild versus fatal = i; moderate versus severe = j, moderate versus critical = k, moderate versus fatal = l; severe versus critical = m, severe versus fatal = n; critical versus fatal = o.

The most common comorbidities in COVID-19 participants were hypertension (n = 64, 33.2%), diabetes mellitus (n = 46, 23.8%), and obesity (n = 31, 16.1%). The most common symptoms were cough (n = 102, 52.8%), dyspnea (n=91, 47.1%), fever (n = 81, 42.0%), myalgia (n = 57, 29.5%), headache (n = 49, 25.4%), asthenia (n = 45, 23.3%), diarrhea (n = 29, 15.0%), anosmia (n = 18, 9.3%), and chest pain (n = 15, 7.8%) (**Table 1**). In our cohort, 86.5% (n=167) were hospitalized for COVID-19; 37.8% (n=73) required intensive care unit (ICU) and only 13.5% (n=26) were not hospitalized. Of the 73 patients in the ICU, 45 (23.3%) required mechanical ventilation for cardiovascular stabilization (**Table 1**).

The hematological and biochemistry parameters are presented in **Table 2**. The data showed marked lymphopenia in the severe, critical, and fatal groups compared to control group and in the fatal group compared to mild and control groups (p<0.05) and a neutrophilia in critical and fatal groups compared to controls, and to the fatal group compared to the mild, moderate and severe groups and in critical compared to severe groups (p<0.05).

**Table 2:** Blood biochemical and hematological parameters of participants in the study (n=220).

Baseline Variable median (IQR)	Control N=27	All patients N=193	Mild N=37	Moderate N=43	Severe N=63	Critical N=14	Fatal N=36	*p* value
Blood cell, median (IQR)								
**Erythrocytes (10^6^/µL)**	4.8 (4.5-5.0)	4.2 (3.4-4.8)	4.5 (3.7-5.2)	4.2 (3.7-4.7)	4.4 (4.0-4.9)	3.8 (3.4-4.1)	3.1 (2.8-4.3)	^d,e,i,l,n^
**Hemoglobin (g/dL)**	14.3 (13.5-14.8)	12.5 (10.5-14.2)	13.4 (11.7-15.2)	12.5 (11.0-14.3)	13.1 (11.9-14.6)	10.9 (10.2-12.7)	9.3 (8.3-12.7)	^d,e,l,n^
**Leucocyte counts (µL)**	6.6 (5.4-7.5)	8.5 (6.0-12.1)	7.3 (5.2-8.6)	8.0 (6.1-11.9)	7.5 (4.8-10.3)	12.7 (8.3-17.8)	12.1 (8.5-16.1)	^d,e,m,n^
**Neutrophil counts (µL)**	3.9 (2.9-4.6)	6.1 (3.7-9.2)	3.7 (2.4-6.4)	5.5 (3.,7-8.0)	5.5 (3.4-8.1)	9.8 (5.8-14.4)	9.3 (6.7-12.8)	^d,e,i,m,l,n^
**Lymphocyte counts (µL)**	2.2 (1.6-2.8)	1.3 (0.8-1.9)	1.9 (1.5-2.2)	1.7 (0.9-2.2)	1.3 (0.8-1.7)	1.3 (0.7-1.8)	0.7 (0.5-1.4)	^c,d,e,i,l^
**Monocyte counts (µL)**	339.0 (189.0-553.5)	413.0 (272.8-703.0)	288.0 (226.5-438.5)	446.0 (281.3-729.0)	399.0 (284.5-618.8)	633.5 (235.5-954.8)	504.0 (281.0-762.0)	^ns^
**Platelet counts (µL)**	231.5 (195.3-285.8)	227.0 (177.1-291.4)	213.8 (152.5-253.7)	227.8 (180.5-285.4)	263.1 (195.0-327.1)	247.6 (188.1-301.5)	175.8 (102.7-233.2)	^n^
Blood biochemistry, median (IQR)								
**D-dimer (mg/L)**	**-**	398.0 (72.4-849.0)	328.0 (149.0-503.8)	369.0 (72.2-653.0)	308.5 (757.3-2783.0)	1107.0 (54.9-4526.0)	571.0 (266.3-1142.0)	^ns^
**C-reactive** **protein (mg/dL)**	0.2 (0.1-0.6)	7.0 (1.7-19.6)	1.7 (0.7-4.4)	7.4 (1.8-20.9)	5.6 (0.9-10.9)	9.3 (5.8-34.3)	11.8 (7.3-25.6)	^a,b,c,d,e,m^
**Ferritin (ng/mL)**	150.0 (94.1-208.0)	671.0 (285.9-1291)	159.2 (42.8-454.4)	522.6 (303.6-1302.0)	522.9 (205.6-831.6)	901.1 (272.1-1474.0)	1180.0 (711.5-2794)	^b,c,d,e,f,i,n^
**Albumin (mg/dL)**	4.3 (4.2-4.4)	3.0 (2.7-3.4)	3.2 (2.8-3.8)	3.3 (3.0-3.7)	3.2 (2.9-3.5)	2.6 (2.4-2.8)	2.6 (2.3-3.1)	^h,k,l,m,n^
**Total bilirubin (mg/dL)**	0.6 (0.4-0.8)	0.5 (0.3-0.7)	0.3 (0.3-0.5)	0.5 (0.3-0.6)	0.5 (0.3-0.6)	0.4 (0.3-0.7)	0.5 (0.3-0.8)	^a,b,c,d,e,k,l,m,n^
**Alanine** **aminotransferase (UI/L)**	18 (14-26)	41 (23-65)	15 (10-52)	33 (17-70)	41 (24-62)	47 (29-89)	47 (23-70)	^b,c,d,e^
**Aspartate aminotransferase (UI/L)**	22 (19-27)	36 (23-54)	18 (13-30)	32 (20-47)	34 (22-47)	49 (26-98)	49 (33-70)	^d,e,i^
**Creatinine (mg/dL)**	0.8 (0.7-1.0)	0.9 (0.7-1.3)	0.6 (0.5-0.7)	0.9 (0.8-1.1)	0.9 (0.7-1.2)	0.8 (0.7-1.5)	1.4 (1.0-2.8)	^e,f,g,i,l,n^

IQR, Interquartile range; ns, not significant. Comparison of the control (healthy participants) with all patients. Categorical variables represented as number (percentage) and compared using Fisher’s exact test. Continuous variables represented as median (interquartile range) and compared using one-way analysis of variance (ANOVA) and Kruskal-Wallis test. *p<0.05. Significance comparing control versus mild = a, control versus moderate = b, control versus severe = c, control versus critical = d, control versus fatal = e; mild versus moderate = f, mild versus severe = g, mild versus critical = h, mild versus fatal = i; moderate versus severe = j, moderate versus critical = k, moderate versus fatal = l; severe versus critical = m, severe versus fatal = n; critical versus fatal = o.

### Levels of anti-SARS-CoV-2 antibodies in the first month post-infection are associated with distinct COVID-19 outcomes

**Figure 1:**
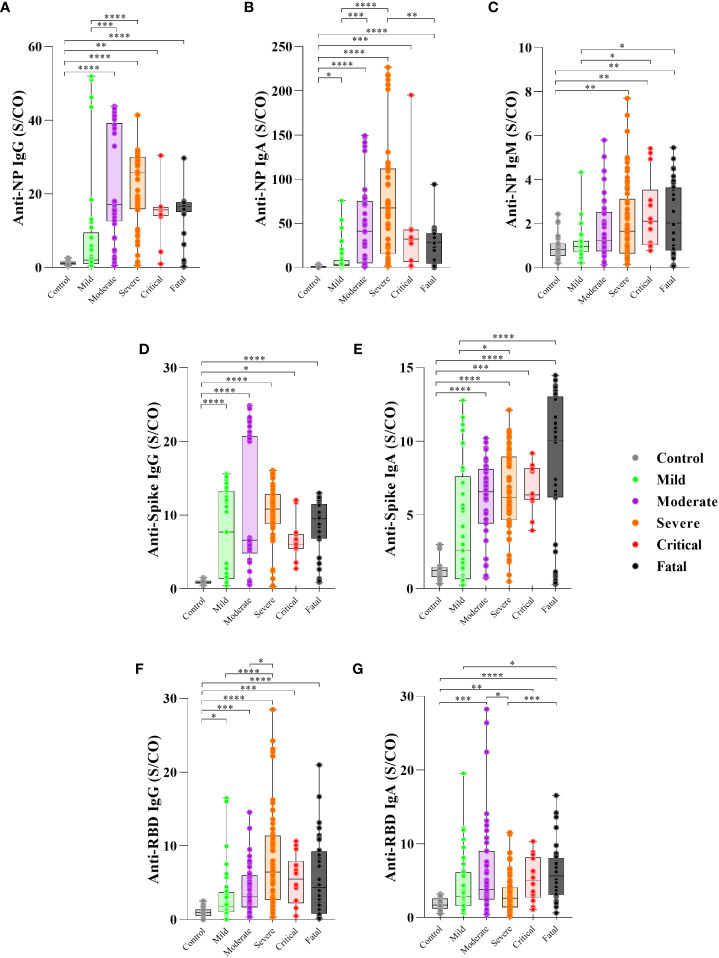
Levels of antibody to SARS-CoV-2 proteins in the first 30 days PSO and disease severity. Serum of patients with different clinical status of COVID-19, (uninfected controls, n = 27; mild, n = 37; moderate, n = 43; severe, n = 63; critical n = 14, fatal, n = 36) was analyzed for the presence of anti-nucleoprotein (NP) IgG **(A)**, IgA **(B)** and IgM **(C)**, anti-Spike IgG **(D)** and IgA **(E)** protein and anti-RBD IgG **(F)** and IgA **(G)** antibodies measured by enzyme-linked immunosorbent assay (ELISA). Results are expressed as the index calculated as the ratio of sample/cutoff OD (S/CO) as described in Methods. Boxes represent the 25th to 75th percentiles. Each dot represents a single individual, with distribution in maximum and minimum values. The line inside the box indicates median values. Kruskal-Wallis test was used for comparison of antibody response between groups. *p< 0.05, **p < 0.01, ***p < 0.001 and ****p <0.0001.

First, we assessed the levels and frequency of seropositivity to SARS-CoV-2-specific antibodies during the first 30 days PSO. Moderate, severe, critical and fatal groups of COVID-19 participants showed higher levels of antibody anti-SARS-CoV-2 proteins in comparison to controls (**Figure 1**), except for anti-NP IgM (control *vs*. moderate, p=0.1236) (**Figure 1C**).

The serum levels of anti-NP IgG antibodies were higher in the moderate (p=0.0002) and severe (p<0.0001) groups than in the mild group (**Figure 1A**) and higher frequency of seropositivity (95% for moderate, 96% for severe) (**Supplementary Figure 1A**).

Anti-NP IgA levels were higher in the severe (p<0.0001) and moderate (p=0.0008) groups than in the mild group, while the fatal group showed lower levels (p=0.0038) than the severe group (**Figure 1B**), with frequency of seropositivity of 100% for severe and critical, 93% for moderate and 86% for fatal group (**Supplementary Figure 1B**). Anti-NP IgM was higher in the critical (p=0.0406) and fatal (p=0.0301) groups compared to the mild group (**Figure 1C**), with frequency of seropositivity 64% for critical and 73% for fatal group (**Supplementary Figure 1C**).

However, regarding the anti-spike specific antibodies, IgG levels showed no significant differences among the COVID-19 groups (p>0.05) (**Figure 1D**) but the frequency of seropositivity was higher in the severe (96%) and critical (100%) groups than in the mild (75%) group (**Supplementary Figure 1D**). Anti-spike IgA levels were higher in the fatal (p<0.0001) and severe groups (p=0.0184) than in the mild group (**Figure 1E**) and higher frequency of seropositivity (85% for fatal, 96% for severe) (**Supplementary Figure 1E**).

The levels of anti-RBD IgG were higher in the severe group than in the mild (p<0.0001) and moderate (p=0.0263) groups (**Figure 1F**) with of 95% frequency of seropositive in severe group (**Supplementary Figure 1F**). In contrast, the severe group presented lower anti-RBD IgA levels than the moderate group (p=0.0342), and the fatal group presented higher anti-RBD IgA levels than the mild (p=0.0347) and severe groups (p=0.0004) (**Figure 1G**), with of 91% frequency of seropositive in the moderate and 95% for fatal group (**Supplementary Figure 1G**). Considering the antibodies detected to the three proteins, all participants produced at least one antibody type to at least one SARS-CoV-2 antigen, except for one participant in the mild group (data not shown).

In order to investigate whether there was a correlation between the SARS-CoV-2 viral copy and antibody isotypes and disease severity, we performed a correlation analysis between Ct values and antibody levels. We considered a correlation when the r is higher than 0.3. The levels of anti-RBD IgG from all COVID-19 patients were positively correlated with SARS-CoV-2 Ct values, which means lower viral load (r=0.3766 p=0.0004) (**Supplementary Figure 2A**). Nonetheless there was no correlation between Ct value and anti-RBD IgA (**Supplementary Figure 2A**), anti-Spike IgG and IgA (**Supplementary Figure 2B**) and anti-NP IgG, IgM, and IgA (r < 0.3) levels (**Supplementary Figure 2C**). Interestingly, regarding severity, in the mild group, it was observed positive correlation between anti-Spike IgG levels and SARS-CoV-2 Ct values (r=0.4368, p=0.0342) (**Supplementary Figure 3A**), although there was no correlation with anti-Spike IgA, and anti-NP IgG, IgA and IgM, anti-RBD IgG and IgA levels with SARS-CoV-2 Ct values (**Supplementary Figures 3A–C**). Moreover, it was observed positive correlation between anti-RBD IgG levels and SARS-CoV-2 Ct value, in the moderate group (r=0.4620, p=0.0265) (**Supplementary Figure 4A**), even though there was no correlation with anti-RBD IgA, anti-NP IgG, IgA and IgM and anti-spike IgG and IgA levels (**Supplementary Figures 4A–C**). Moreover, there was a positive correlation between SARS-CoV-2 Ct value and anti-NP IgM and anti-RBD IgG levels in the severe plus critical groups (r=0.5189 p=0.0039) (**Supplementary Figures 5A, B**), but not with anti-NP IgG and IgA, anti-RBD IgA and anti-Spike IgG and IgA (**Supplementary Figures 5A–C**). Nevertheless, we found no correlation between viral load and anti-SARS-CoV-2 antibodies detected in the fatal group (p>0.05) (**Supplementary Figure 6**).

Additionally, we have detected several cytokines in the plasma of COVID-19 patients in in different outcomes in our previous study with the same cohort (29). We then analyzed if there was a correlation between the levels of antibodies against proteins of SARS-CoV-2 and the cytokine production (**Supplementary Figures 7**–**10**). Taken all COVID-19 patients in the acute phase, we detected a positive correlation between IL-6 and anti-Spike IgA levels (r=0.3517, p=0.0004) (**Supplementary Figure 7A**), and anti-RBD IgA levels (r=0.3247, p=0.0010) (**Supplementary Figure 7B**), although there was no correlation between IL-6 and anti-Spike IgG, anti-RBD IgG and anti-NP IgG, IgA, IgM levels (**Supplementary Figures 7A–C**). In contrast, regarding severity, there was a negative correlation between IL-6 and anti-NP IgG (r=-0.4523, p=0.0232) and IgA (r=-0.4626, p=0.0228) levels in the severe plus critical group (**Supplementary Figure 8A**), nevertheless, there was no correlation between IL-6 anti-NP IgM, anti-Spike and anti-RBD IgG, IgA (**Supplementary Figures 8A–C**). Additionally, the analysis of IL-2 (**Supplementary Figure 9**) showed a positive correlation with anti-NP IgG levels (r=0.3182, p=0.0014) (**Supplementary Figure 9A**) in all the COVID-19 patients, however, there was no correlation between IL-2 and anti-NP IgA, IgM, anti-Spike and anti-RBD IgG, IgA levels (**Supplementary Figures 9A–C**). In the fatal group, we found a negative correlation between IL-2 and anti-Spike IgA levels (r=-0.4253, p=0.0383) (**Supplementary Figure 10A**). In contrast, there was no correlation between IL-2 and anti-Spike IgG, anti-NP IgG, IgA, IgM and anti-RBD IgG, IgA levels (**Supplementary Figures 10A–C**). The analysis of other cytokines such as IL-10, IL-4, IFN-γ, and TNF-α did not show any significant correlation with antibody levels against SARS-CoV- 2 proteins (data not shown).

### The neutralizing antibody responses to SARS-CoV-2 across the clinical spectrum of COVID-19

**Figure 2:**
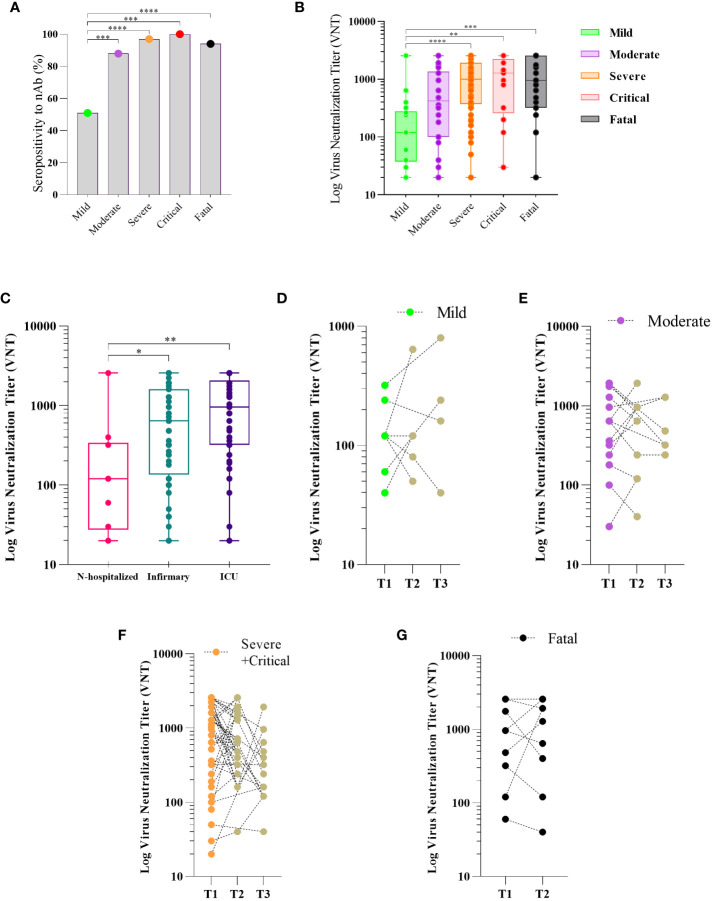
Neutralizing antibody response to SARS-CoV-2 and disease severity COVID-19. Sera of patients with different clinical status of COVID-19, (mild, n = 18; moderate, n = 34; severe, n = 60; critical n = 13, fatal, n = 30) were analyzed for the presence of neutralizing antibody (nAb). Maximum neutralization titer was measured by Virus Neutralizing Titers (VNT) and results are expressed as log of VNT as described in Methods. Frequency of positivity to nAb **(A)** and VNT levels **(B)** in the first 30 days after the onset of symptoms (PSO) in the different clinical status of the disease. Boxes represent the 25th to 75th percentiles and each dot represents a single individual, with distribution in maximum and minimum values. The line inside the box indicates median values. nAb response according to hospital care in COVID-19 patients **(C)**. Kinetics of Nab response to SARS-Cov-2 in three periods of time PSO: T1 (≤ 30 days), T2 (>30 and <180 days) and T3 (≥ 180 days) for patients classified as mild **(D)**, moderate **(E)**, severe plus critical **(F)**, and two timepoints (T1 (≤ 30 days) and T2 (>30 and < 60 days) for those who fatal **(G)**. Each dot represents the antibody response of a single individual in different periods of time linked by the dotted line. Fisher’s exact test was used for comparison of frequency of nAb response **(A)**, Kruskal-Wallis test was used for comparison of nAb level response **(B, C)**. Wilcoxon matched-pairs test was used for comparison of antibody response among groups **(D–F)**. *p< 0.05, **p < 0.01, ***p < 0.001 and ****p <0.0001.

We determined the nAb levels in the serum of SARS-CoV-2 infected individuals throughout the clinical course of the infection. We first assessed the levels and frequency of nAb in the first 30 days of PSO (time point T1). We found nAb seropositivity of 81% (data not shown) among COVID-19 individuals. The frequency of nAb detection was significantly higher in participants who developed moderate, severe, critical, and fatal COVID-19 than in those who developed mild disease (**Figure 2A**). Moreover, higher levels of nAb were observed in individuals who progressed to severe and critical disease and in the fatal group compared to the mild group (*vs.* severe p<0.0001, critical p=0.0075, fatal p=0.0009), no differences among severe, critical, and fatal groups compared to the moderate group (*vs.* severe p=0.1258, critical p=0.8771, fatal p=0.4778) (**Figure 2B**). Additionally, COVID-19 participants who were hospitalized had higher nAb titers compared to those who were not (*vs.* infirmary p=0.04, ICU p=0.002) (**Figure 2C**). However, no significant difference in nAb levels was observed between ICU and infirmary admitted participants (p=0.1654) (**Figure 2C**). Longitudinal follow-up of a subset of individuals in each group of the clinical outcome, between 1 and 6 months PSO (time point 2) and over 6 months PSO (time point 3) did not show significant differences in nAb levels between T1, T2, and T3 time points in any of the COVID-19 groups: mild (**Figure 2D**), moderate (**Figure 2E**), and severe plus critical (p>0.05) (**Figure 2F**). Most of the critical individuals died, thereby, the number of people in this group recruited as recollects was very limited. Thus, due to the scarcity of this group, for follow-up analyses, samples of individuals classified as severe and critical were analyzed together in a single group. The levels of nAbs in the fatal group were not different at the 2 time points analyzed (**Figure 2G**).

### Dynamics of circulating antibodies to SARS-CoV-2 proteins: a longitudinal analysis

Thereafter, we assessed the dynamics of antibody levels longitudinally in a subset of 82 patients at three PSO time points: T1: up to 30 days PSO, T2: 1–6 months PSO, and T3: more than 6 months PSO (only 35 participants for T3).

**Figure 3:**
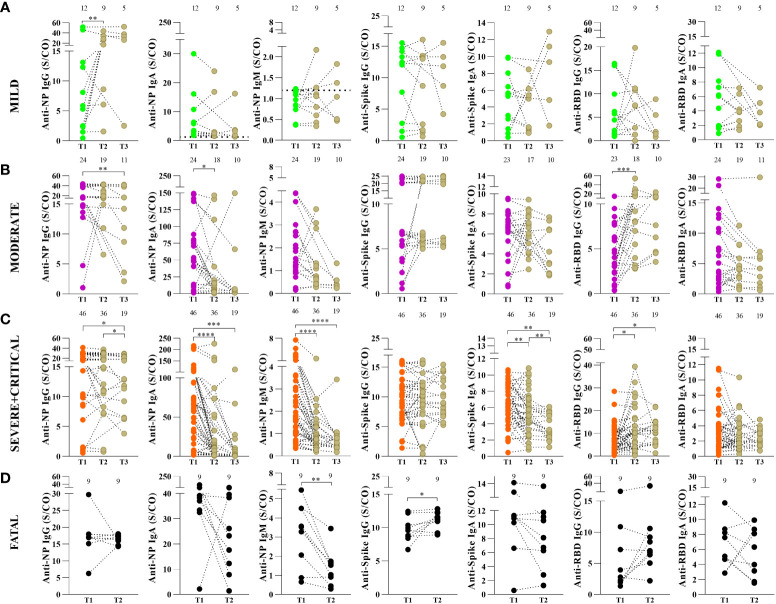
Kinetics of antibody response to SARS-CoV-2 proteins in COVID-19 patients according to disease severity. Anti-nucleoprotein (NP) IgG, IgA and IgM, anti-spike IgG and IgA protein and IgG, anti-RBD IgA and IgM antibodies in mild **(A)**, moderate **(B)** and severe plus critical patients **(C)** were measured by enzyme-linked immunosorbent assay (ELISA) as described in Methods. Serum samples were collected in three periods of time after the onset of symptoms: T1 (≤ 30 days), T2 (>30 and <180 days) and T3 (≥ 180 days). Fatal patients **(D)** were analyzed in two timepoints after the onset of symptoms: T1 (≤ 30 days) and T2 (>30 and < 60 days). Results are expressed as sample/cutoff OD (S/CO). The values above the graph are the numbers of patients in each time point. Each dot represents the antibody response of a single individual in the three periods of time linked by the dotted line. Wilcoxon matched-pairs test was used for comparison of antibody response among the different periods of time. *p< 0.05, **p < 0.01, ***p < 0.001 and ****p <0.0001.

In the mild group, the levels of anti-NP IgG antibodies increased in T2 (*vs*. T1, p=0.004), but the levels of anti-NP IgA and IgM, anti-spike and anti-RBD IgG and IgA showed no significant differences (*vs*. T2, T3, p>0.050) over time (**Figure 3A**), suggesting maintenance of the levels over the time.

In the moderate group, anti-NP IgG antibody levels decreased in T3 (*vs*. T1, p=0.004) and T2 anti-NP IgA (*vs*. T1, p=0.020). In contrast, anti-RBD IgG levels increased in T2 (*vs*. T1, p=0.001), while the levels of anti-NP IgM, anti-spike, and anti-RBD IgA did not show significant differences (*vs*. T2, T3, p>0.050) over time (**Figure 3B**).

For the severe and critical groups analyzed together, we observed a decrease in anti-NP IgG levels in T3 (*vs*. T1, p=0.0289, and *vs*. T2, p=0.0391), IgA levels were lower in T2 (*vs*. T1, p<0.0001) and T3 (*vs*. T1, p=0.0002) and similarly observed for IgM were lower in T2 (*vs*. T1, p<0.0001) and T3 (*vs*. T1, p=0.0001). Moreover, anti-spike IgA was lower in T2 (*vs*. T1, p=0.0063) and T3 (*vs*. T1, p=0.0033 and *vs*. T2, p= 0.0078). The anti-RBD IgG was higher in T2 (*vs*. T1, p=0.0105) and T3 (*vs*. T1, p= 0.0361). The levels of anti-spike IgG and anti-RBD IgA showed no significant differences over time in the severe + critical group (*vs*. T2, T3, p>0.050) (**Figure 3C**).

The dynamics at follow-up (T1: ≥30 days PSO to T2: ≥60 days PSO) in the fatal group showed that anti-spike IgG antibody levels increased (p=0.0273), while anti-NP IgM levels decreased (p=0.0078) between T1 to T2. Regarding the follow-up of individuals displaying fatal outcomes, the levels of anti-NP IgG and IgA, anti-spike IgA and anti-RBD IgG, and IgA showed no significant difference between the T1 and T2 time points (p=0.7344) (**Figure 3D**).

### Lower levels of IgG and IgA anti-NP and higher IgA anti-spike and anti-RBD discriminate survival *versus* fatal in COVID-19

**Figure 4:**
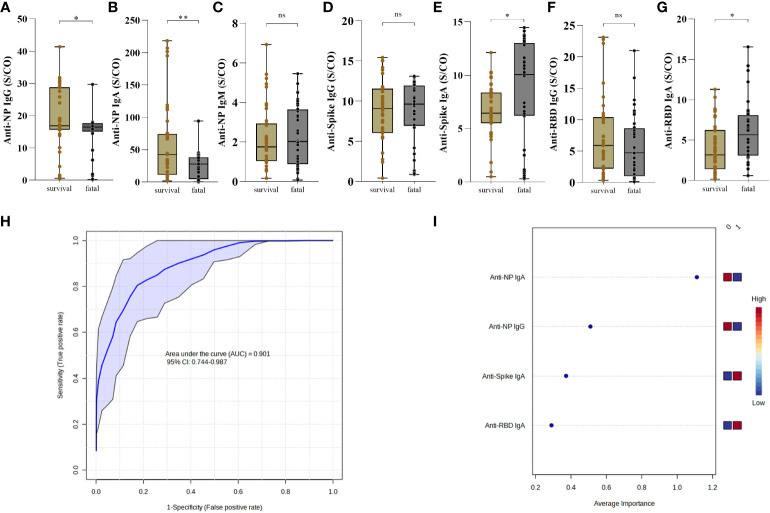
Antibody response to SARS-CoV-2 proteins distinguish survival and fatal patients with COVID-19. The presence of anti-NP IgG **(A)** IgA **(B)** and IgM **(C)**, anti-Spike IgG **(D)** and IgA **(E)** and anti-RBD IgG **(F)** and IgA **(G)** were analyzed in the first 30 days post-symptoms onset. Antibodies in the serum of patients who survived (n = 37) and those who died (n = 36) was measured by enzyme-linked immunosorbent assay (ELISA). Results are expressed as sample/cutoff OD (S/CO) as described in Methods. Boxes represent the 25th to 75th percentiles. Each dot represents a single individual, with distribution in maximum and minimum values. The line inside the box indicates median values. Mann-Whitney test was used for comparison of antibody response between groups. Statistical significances are shown as *p < 0.05, **p < 0.01. Receiver Operating Characteristic curve showing sensitivity versus specificity for discrimination of survival and fatal individuals were derived for each combination of the high performing for anti-NP IgG and IgA, anti-Spike and anti-RBD IgA (ROC AUC=0.879, blue line), is presented with 95% confidence intervals (shown in the blue regions) **(H)**. The most discriminating antibody are shown in descending order of their coefficient scores. The color boxes indicate whether antibody concentration is increased (red) or decreased (blue) in [0] survival vs [1] fatal **(I)**.

Considering only individuals admitted to the ICU, we classified them into survivors and fatal individuals and compared the frequency and levels of SARS-CoV-2 antibody production at the first 30 days PSO. The fatal group presented lower levels of anti-NP IgG (p=0.0139) (**Figure 4A**), lower levels of anti-NP IgA antibodies (p=0.0014) (**Figure 4B**), but no differences of anti-NP IgM (p=0.7439) (**Figure 4C**) compared to the survival group. Regarding the antibody levels to spike protein, there was no difference related to IgG (p=0.5016) (**Figure 4D**), but interestingly the fatal group presented significant higher levels of IgA antibody levels (p=0.0131) compared to survival (**Figure 4E**). Moreover, there were also no different levels of anti-RBD IgG (p=0.2803) (**Figure 4F**), but remarkably there was a significant increase of anti-RBD IgA antibodies, in the fatal group compared to the survival (p=0.0052) (**Figure 4G**).

Concerning the frequency of individuals with positive antibody response to NP protein there was no difference of IgG (p=0.3505) (**Supplementary Figure 11A**), but it was observed a significant increase of IgA (p=0.0232) (**Supplementary Figure 11B**) in the survival compared to the fatal group. In addition, there was no difference in the frequency of anti-NP IgM (p=0.3342) between the groups (**Supplementary Figure 11C**). A lower frequency of individuals with anti-spike IgG in the fatal group (p=0.0251) (**Supplementary Figure 11D**), although there was no difference of IgA (p>0.9999) between the groups (**Supplementary Figure 11E**). In relation to RBD, there was no difference in the frequencies of individuals producing IgG (p=0.0956) and IgA (p=0.0996) (**Supplementary Figures 11F, G**).

To determine whether the observed differences discriminate individuals who died from those who survived COVID-19, we used anti-NP IgG and IgA, anti-Spike and anti-RBD IgA measurements as input in Receiver Operating Characteristic (ROC). The ROC analysis resulted in an Area Under the Curve (AUC) = 0.901, demonstrating a strong ability of these antibody features to predict fatal outcomes of COVID-19 (**Figure 4H**). Average importance is given as anti-NP IgA (lower) > anti-NP IgG (lower) > anti-Spike IgA (higher) > anti-RBD IgA (higher) in fatal outcome (**Figure 4I**).

Moreover, we observed no difference, in time point 2 (T2: >30 and < 60 days) in the levels of anti-NP IgG (p=0.7051) (**Supplementary Figure 12A**), IgA (p=0.6098) (**Supplementary Figure 12B**) and IgM (p=0.4634) (**Supplementary Figure 12C**). The levels were higher of anti-Spike IgG (p=0.0427) (**Supplementary Figure 12D**), although there was no difference about the anti-Spike IgA (p=0.2538) compared to the survivors (**Supplementary Figure 12E**). Also, no difference about the levels of anti-RBD IgG (p=0.8307) (**Supplementary Figure 12F**), but levels were higher of anti-RBD IgA (p=0.0044) (**Supplementary Figure 12G**). We also compared the levels of antibodies in fatal and survivors in two age groups: 40 to 59 years and ≥60 years. We noted that the levels of anti-NP IgM (p=0.0039), anti-Spike (p=0.0221) and anti-RBD IgA (p=0.0006) were higher in fatal participants with 40–59, although no difference about the levels of anti-NP IgG (p=0.3863) and IgA (p=0.1469), anti-spike IgG (p=0.6518o) and anti-RBD IgG (p=0.9263) (**Supplementary Figure 13A**). However, the levels of anti-NP IgG (p=0.0321) and IgA (p=0.0204) were lower in participants with ≥60 years of age (**Supplementary Figure 13B**) who died but was no difference about the levels of anti-NP IgM (p=0.1976), anti-Spike IgG (p=0.1750) and IgA (p=0.0959), and anti-RBD IgG (p=0.1446) and IgA (p=0.4339).

### Correlation of antibody levels of SARS-CoV-2 and blood parameters

**Figure 5:**
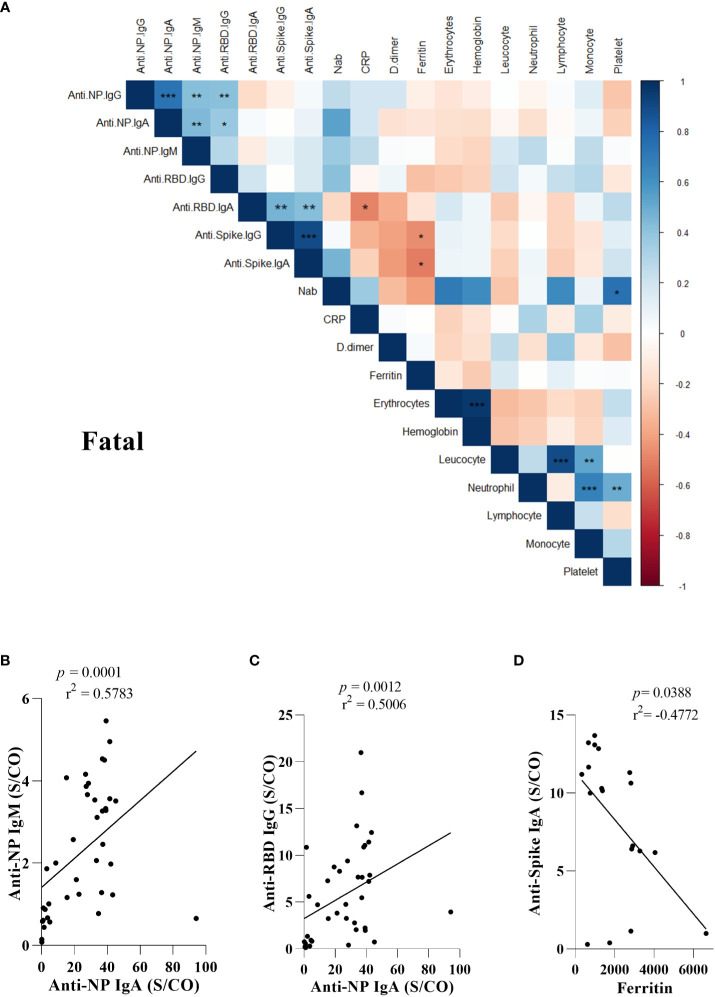
Antibody response to SARS-CoV-2 proteins and mortality in COVID-19. Correlations among lymphocyte, monocytes, neutrophil, platelet, erythrocyte, hemoglobin, D-dimer, PCR, Ferritin, and antibodies to SARS-CoV-2 proteins **(A)**. Correlation matrix plot of all variables to identify potential inter-variables correlations. Spearman R values are shown from red (−1.0) to blue (1.0). Only boxes showing a significant correlation and r > 0.5 have been highlighted. Blank fields indicate lack of signal. Hb., Hemoglobin; Neutro., Neutrophil; Lympho. Lymphocyte; Mono., Monocyte; PSO., post-symptoms onset; TAP., partial thromboplastin time; TTPA., activated partial thromboplastin time; CRP., C-reactive protein; ALT., Alanine aminotransferase; AST., Aspartate aminotransferase; nAb. Neutralizing antibody. Spearman correlation between each subclass of specific antibody response to nucleoprotein (NP), Spike and RBD SARS-Cov-2 proteins was analyzed by non-linear regression and those with significant *p* values are shown **(B, C)** and the correlation between anti-Spike IgA and ferritin **(D)**. The presence of antibodies in the serum of patients who died was measured by enzyme-linked immunosorbent assay (ELISA). Statistical significances are shown as *p< 0.05, **p < 0.01, ***p < 0.001.

To determine whether there were correlations between routine blood and biochemical data with antibody levels, we performed analysis using a correlogram, including all COVID-19 participants (**Supplementary Figure 14**): mild (**Supplementary Figure 15A**), moderate (**Supplementary Figure 15B**), severe (**Supplementary Figure 15C**), critical (**Supplementary Figure 15D**), and fatal groups (**Figure 5A**).

In the fatal group, we observed positive correlations between positive anti-NP IgG *vs* IgA, IgM and anti-RBD IgG, anti-NP IgA *vs* IgM and IgG RBD, anti-Spike IgG *vs* IgA, platelets *vs* nAb, erythrocytes *vs* hemoglobin, leukocytes *vs* lymphocytes and monocytes, neutrophils *vs* monocytes and platelets. Additionally, we noted a negative correlation between Ferritin vs anti-Spike IgG and IgA (**Figure 5A**).

Among the correlations, we investigated exclusively in the fatal group, the positive correlations between the levels of anti-NP IgA and IgM (p = 0.0001, r^2^ = 0.5783) and between anti-NP IgA and anti-RBD IgG levels (p = 0.0012, r^2^ = 0.5006) (**Figures 5B, C**). In addition, we investigated potential correlations between antibody levels and those of inflammatory markers, such as C-reactive protein, D-dimer, and ferritin. Ferritin showed higher expression in the fatal group (*vs*. severe, mild, p = >0.05) and a negative correlation (p = 0.0388, r^2^ = -0.4772) with anti-Spike IgA (**Figure 5D**).

No correlation was observed between other laboratory data and the antibody levels (**Supplementary Figure 14**). Regarding peripheral blood cell count, we found positive correlations between erythrocytes, hemoglobin, leukocytes, neutrophils, and monocytes in the moderate, severe, and critical groups (**Supplementary Figures 15B–D**). Concerning biochemical laboratory parameters, D-dimer showed a positive correlation with the levels of anti-NP antibodies, C-reactive protein, and anti-RBD IgA in the mild group (**Supplementary Figure 15A**).

## Discussion

In this study, we investigated the dynamics of the development of SARS-CoV-2-specific antibodies and nAb activity to the wild type of virus in a cohort of unvaccinated COVID-19 patients in Brazil, up to 13 months post-infection and the association with clinical outcomes. The results demonstrated that in general COVID-19 groups showed higher levels of antibodies to SARS-CoV-2 antigens compared to the control group, and the antibody levels increase in the spectrum of more severe disease. confirming that SARS-CoV-2 infection induces a robust humoral immune response according to disease severity. Furthermore, we observed higher levels of nAb in the severe, critical, and fatal COVID-19 groups compared to mild group.

Analyzing the SARS-CoV-2-specific antibodies levels, our results showed that the decreased levels of antibodies and lower frequency of seropositivity in the mild group could be related to the short time of infection, lower viral load, and lower inflammatory response, as well as lower antigen exposure compared to the severe and critical groups (30, 31). Considering the severity of COVID-19, anti-NP IgG and IgA and anti-RBD IgG levels were higher in severe group, anti-NP IgM and anti-spike IgA were higher in the fatal groups. Studies have also reported that higher antibody levels, such as anti-NP IgA (32), anti-Spike, and anti-RBD IgG, were associated with illness progression of COVID-19 between 14 to 42 days PSO (33–35). Our results showed a humoral response mediated by a diversity of antibody isotypes to distinct proteins. The hyperinflammatory state in COVID-19, with exacerbated production of cytokines promotes the class-switching of memory B cells to IgG via IFN-γ or IgA via TGF-β, resulting in a variety of antibodies with distinct kinetics (36) which may be an explanation for the production of different anti-SARS-CoV-2 isotypes. In patients with COVID-19, anti-Spike and anti-RBD IgA levels were positively correlated with the inflammatory cytokine IL-6. This cytokine has been shown to favor an isotype class switching of mucosal B cells to IgA (37, 38). In this context, the high production of IL-6 in severe patients could promote the increase of IgA, which can corroborate with the inflammatory profile and may contribute to the role of IgA in the pathogenesis in severe cases. In contrast, anti-NP IgG levels were positively correlated with the IL-2 levels, which may promote IgG isotype switching (39, 40). Considering, the severe plus critical group, IL-6 was negatively correlated with anti-NP IgG and IgA levels, and in the fatal group IL-2 was negatively correlated with anti-Spike IgA. Taken together, the results suggest that cytokine production could influence the anti-SARS-CoV-2 antibody isotype switching and in the clinical outcomes, although further studies are necessary to clarify this point.

We observed increase of anti-Spike and anti-RBD IgA levels in fatal group compared to survival group. The association of IgA with disease severity has been shown in the literature (41). For instance, anti-S1 IgA has also been associated with worse clinical evolution (36, 41), suggesting that SARS-CoV-2 infection may be at least in part an IgA-mediated disease since IgA in serum can deposit on tissues, causing damage in several organs, which is a common event in severe cases of COVID-19 (42). Although mucosal IgA was not measured, we do not discard its role in the severity of the disease, as described by Ruiz et al. (43), who showed the persistence of anti-S1 and anti-RBD IgA and the presence of immune complexes in bronchoalveolar lavage in individuals who died, reinforcing the contribution of IgA immune complexes to the immunopathogenesis of SARS-CoV-2 infection (43). Moreover, the crosslink of IgA bound to pathogen antigen with FcRα enhances the signaling cascades, culminating in the increase of inflammation and potentiation of the immune responses that can be protective or detrimental (44). Furthermore, the presence of serum IgA has been suggested as a biomarker for severe COVID-19 (45). Several explanations can rise for the possible pathogenic role of IgA (43, 44, 46). Since IgA are produced against different proteins and may be associated with distinct stages of diseases, our data cannot allow us to link IgA isotype as a deleterious role. Further studies, including antibody subclass function, are need clarify this role of IgA in COVID-19 (47–49).

In the virus neutralization assay, the gold standard assay for the detection of nAbs, a marked presence of nAbs was observed in all groups, but only the mild and moderate groups showed lower levels of nAbs when compared to other groups. Different from our data, Lucas et al. (2021) observed in the first 14 days of PSO that patients who did not present nAb levels progressed to death compared to the other groups according to severity disease, reinforcing that the early production of nAb are associated with survival outcome (50). Our results do not allow us to infer whether the early production of nAbs guided the clinical outcome, since we usually had blood sampling collection at different days PSO. The nAb detected in the individuals in our study might promote virus neutralization in different stages of COVID-19. The overall differential antibody profile found in the fatal group consisted of higher levels and frequency of seropositivity of IgM anti-NP and IgA anti-spike, but lower levels and frequency of detection of IgA anti-NP antibodies and lower frequency of detection of IgG anti-RBD compared to the severe group. Similar to all other clinical groups, the fatal group presented a higher frequency and titers of nAbs, only compared to the mild COVID-19 group. Moreover, we showed a positive correlation of nAb with anti-RBD IgG antibody levels only in the severe and critical groups. In contrast, we showed a significance positive correlation between nAb levels and anti-Spike IgG antibody just in the moderate group. The longitudinal dynamics of nAbs in our cohort showed no significant changes in the different groups over time, suggesting a long-term maintenance of the nAbs for a long time. In relation to this, it was shown that nAbs can persist up to 18 months in patients who had mild COVID-19 (51). In addition, it has been shown that neutralizing activity may decrease after four months (52) or present lower potency in severely ill patients (33). Even though, there are severe, critical and fatal individuals in our cohort, the nAbs levels were present in those patients.

When we investigated the persistence of humoral immunity, in the follow-up analysis of 6 months or more, we observed that anti-spike IgA levels were decreased in the severe plus critical groups compared to the first ≤30 days PSO (T1). These findings are in agreement with those observed by Fedele et al., who demonstrated that there was no decline in IgG levels but that anti-spike IgA decreased after 6 months of infection in mild/moderate and severe groups (53). In fact, humoral response kinetics revealed maintenance of levels in the mild group, with gradual reduction in moderate and severe plus critical groups, revealing loss of this maintenance of anti-NP antibodies, but with stability in the production of anti-spike and anti-RBD IgG antibodies. In the longitudinal antibody levels in the severe + critical group, we must consider that this robust result be related to the number of individuals recruited for another collection of blood in the recovery phase.

Regarding the outcome of SARS-CoV-2 infection, ≤30 days PSO (T1), patients with COVID-19 admitted to the ICU, our analyses revealed a distinct pattern of antibody production, with higher levels of anti-NP IgG and IgA in the survival group, but anti-Spike and anti-RBD IgA had higher levels in the fatal group. In this context, it has been shown that increased levels of antibody in severe individuals may be related to the higher viral load and longer exposure to the virus in severe patients, suggesting that a higher viral load may induce greater antibody production (54–56). Considering that viremia decreases exponentially with the linear increase in Ct values obtained by RT-PCR, when we evaluated the Ct for SARS-CoV-2 and the antibody levels, we saw that the lower is the viremia, the higher are the anti-RBD IgG levels, the same occurring in the moderate and severe plus critical groups. A possible explanation for the control of viremia is the neutralization capacity of anti-RBD IgG and other antibody effector functions via Fc receptors described previously (57). A recent study performed with COVID-19 patients demonstrated that increased anti-spike IgG antibody levels were associated with the worst disease, suggesting that this profile can be explained by antibody-mediated immunopathology (50). The mechanism underling the association of high levels of anti-Spike and anti-RBD with severity and death should be further investigated.

Antibody levels, according to age, showed that individuals with 40-59 years produced higher levels of anti-NP IgM, anti-RBD and anti-spike IgA and individuals with 60 years or more showed less anti-NP IgG and IgA in the fatal group. Age is associated with an increase in the number of people who become seriously ill or die from COVID-19 since older people start to have complications from COVID-19 (53). Immunosenescence is associated with a reduced immune response capacity, either by dysfunction of the innate immune response, increase in inflammatory cytokines, or deficiency in the production of B cells; the repertoire of T-cell receptors are limited, and regulatory T-cells (Treg) are further efficient, among other changes in the distribution of immune cells (58–61).

The longitudinal analysis of the fatal group in the period between 30 and 60 days PSO showed a decrease in the levels of anti-NP IgM and an increase in anti-spike IgG levels. In general, the increase in the IgG isotype and the reduction in IgM is due to the function of B lymphocytes after interaction with the virus, and later, the exchange of the IgM isotype for IgG, with a consequent production of low-binding plasma cell antibodies (62), which is a common event in infections, especially in the acute phase. A correlation test was performed on inflammatory markers, such as D-dimer, CRP, and ferritin, and we found a negative correlation between anti-spike IgA and ferritin, one of the parameters associated with worsening clinical progression. Increased ferritin synthesis is regulated by the production of pro-inflammatory cytokines or by the extravasation of intracellular ferritin caused by cellular damage (63). Studies reported existence the ferritin positive correlations with anti-Spike IgG, and their increased ratio in severe cases (50, 64), further investigation is necessary to clarify the role of serum ferritin levels with antibodies in the pathogenesis of COVID-19.

A positive correlation of anti-NP IgA with anti-NP IgM and anti-NP IgA with anti-RBD IgG in the blood of fatal patients with COVID-19 was detected. Interestingly, comparison to mild disease presentation, though in the fatal group, levels of anti-NP IgA significantly decreased in comparison to severe COVID-19, suggesting that this could be a relevant indication of disease aggravation and evolution to death. In this context, it has been shown that the antibody anti-NP is more sensitive, conserved, and stable and appears in the first days after the onset of symptoms. Antibodies against NP protein, one of the four structural proteins and main sign for the virus infection, have been used to detect early infection (65). Furthermore, the presence of anti-NP antibodies in the sera of patients has been associated with disease severity (65–68). A possible mechanism that explains this association is that the anti-NP antibody via the Fc-receptor induces the production of the main pro-inflammatory cytokine, IL-6, in lung alveoli infected with SARS-CoV-2 due to the cytokine storm observed in COVID-19, potentiating the disease severity (67, 69).

Our study has some limitations that may explain the differences observed in relation to other studies, as we do not have samples from the first 10 to 15 days after the onset of symptoms from all patients as a definition of the viral shedding duration. The collection of blood samples was late, most donors had specimens collected 14 days after symptom onset, since these patients were mainly treated at the primary health care level and moved to secondary care hospital where the samples were collected. Another point is related to the low number of critical patients and recovered individual. Even though there those limitations, it was possible to study the humoral immune response in acute phase and longitudinally in distinct groups of patients.

In conclusion, we found that higher levels of anti-NP IgM, IgA, and IgG antibodies, as well as anti-spike IgA and anti-RBD IgG, were associated with worse clinical outcome compared the mild disease, suggesting a potentially deleterious effect when these antibodies are found in excess. On the other hand, we found that higher levels of anti-Spike and anti-RBD IgA, and lower levels of anti-NP IgG and IgA characterize fatal outcomes, suggesting these antibody features as predictors of death from COVID-19.

Future studies should be carried out to determine the ability and the mechanism by which antibodies act against the circulating virus and its role in viral infection and/or replication, providing information about antibodies that protect against reinfection or induce clinical worsening.

## Data availability statement

The original contributions presented in the study are included in the article/**Supplementary Material**; further inquiries can be directed to the corresponding author/s.

## Ethics statement

The studies involving humans were approved by Ethical Appreciation (CAAE: 30804220.2.0000.5078). The studies were conducted in accordance with the local legislation and institutional requirements. The participants provided their written informed consent to participate in this study.

## Author contributions

Conceived and supervised the study: SGF, CPS, MS-F. Recruited participants, collected samples, epidemiological and clinical data: CPS, LCM, AOG, MASBB, JMMS, ARG-J, MHAL, BGNM, SMS, LEX. Processed samples: CPS, LCM, JMMS, ARG-J. Performed and supervised RT–qPCR analysis: DCCA, FSF, MS. Performed and supervised Elisa: CPS, LCM, MS-F, SGF. Performed and supervised Neutralizing antibody assay: VFB, SACJ, ELD, DCAO. Production of RBD protein: JVB-C, AMM. Performed data analysis and generated figures and tables: CPS, MS-F, SGF. Original draft: CPS, SGF, MS-F. Critical discussion, review and editing of the manuscript: SGF, CPS, VC, MS-F, LCM, DCCA, FSF, MS, AOG, MASBB, SACJ, VFB, PRTR, JVB-C, AMM, ALB, IAHP, NLC, VCRF, LGG. Funding acquisition: SGF. All authors reviewed and approved the manuscript.

## Supplementary material

Supplementary Figure 1 Seropositivity to SARS-CoV-2 proteins in COVID-19 patients according to disease severity. Patients with different clinical status were analyzed in the first 30 days post-symptoms onset (mild, n = 37; moderate, n = 43; severe, n = 63; critical n = 13, fatal, n = 36). Detection of anti-nucleoprotein (NP) IgG **(A)**, IgA **(B)** and IgM **(C)**, anti-Spike IgG **(D)** and IgA **(E)** protein and anti-RBD IgG **(F)** and IgA **(G)** antibodies were measured by enzyme-linked immunosorbent assay (ELISA) as described in Methods. Results are expressed as frequency of positive response considered with index > 1.2 calculated as the ratio of sample/cutoff OD (S/CO). Fisher’s exact test was used for comparison of antibody response between groups. *p< 0.05, **p < 0.01, ***p < 0.001 and ****p <0.0001.

Supplementary Figure 2 Ct values reflect and antibodies levels to SARS-CoV-2 proteins. Spearman correlation and non-linear regression (line) were performed between anti-RBD IgG and IgA **(A),** anti-Spike IgG and IgA **(B),** and anti-nucleoprotein (NP) IgG, IgA and IgM **(C),** and with Ct value, performed by RT-PCR. The presence of antibodies in the patients’ (each point) serum was measured by enzyme-linked immunosorbent assay (ELISA). Spearman R and p values are included each one graphic.

Supplementary Figure 3 Ct values reflect and antibodies levels to SARS-CoV-2 proteins in the mild group. Spearman correlation and non-linear regression (line) were performed between anti-Spike IgG and IgA **(A),** anti-nucleoprotein (NP) IgG, IgA and IgM **(B),** and anti-RBD IgG and IgA **(C)** with Ct value, performed by RT-PCR, in the mild group. The presence of antibodies in the patients’ (each point) serum was measured by enzyme-linked immunosorbent assay (ELISA). Spearman R and p values are included each one graphic.

Supplementary Figure 4 Ct values reflect and antibodies levels to SARS-CoV-2 proteins in the moderate group. Spearman correlation and non-linear regression (line) were performed between anti-RBD IgG and IgA **(A),** anti-nucleoprotein (NP) IgG, IgA and IgM **(B),** and anti-Spike IgG and IgA **(C)** with Ct value, performed by RT-PCR, in the moderate group. The presence of antibodies in the patients’ (each point) serum was measured by enzyme-linked immunosorbent assay (ELISA). Spearman R and p values are included each one graphic.

Supplementary Figure 5 Ct values reflect and antibodies levels to SARS-CoV-2 proteins in the severe plus critical group. Spearman correlation and non-linear regression (line) were performed between anti-nucleoprotein (NP) IgG, IgA and IgM **(A),** anti-RBD IgG and IgA **(B),** and anti-Spike IgG and IgA **(C)** with Ct value, performed by RT-PCR, in the severe plus critical group. The presence of antibodies in the patients’ (each point) serum was measured by enzyme-linked immunosorbent assay (ELISA). Spearman R and p values are included each one graphic.

Supplementary Figure 6 Ct values reflect and antibodies levels to SARS-CoV-2 proteins in the fatal group. Spearman correlation and non-linear regression (line) were performed between anti-nucleoprotein (NP) IgG, IgA and IgM **(A),** anti-Spike IgG and IgA **(B),** and anti-RBD IgG and IgA **(C)** with Ct value, performed by RT-PCR, in the fatal group. The presence of antibodies in the patients’ (each point) serum was measured by enzyme-linked immunosorbent assay (ELISA). Spearman R and p values are indicated for each isotype.

Supplementary Figure 7 Correlation of IL-6 with antibodies levels to SARS-CoV-2 proteins. Spearman correlation and non-linear regression (line) were performed between anti-Spike IgG and IgA **(A)**, anti-RBD IgG and IgA **(B)**, and anti-nucleoprotein (NP) IgG, IgA and IgM **(C)** antibodies and IL-6 MFI level values (log scale), in the several plus critical group. Concentration of IL-6 was detected by CBA (28). The presence of antibodies in the serum of patients was measured by enzyme-linked immunosorbent assay (ELISA). Spearman R and p values indicated for each isotype. MFI indicates average of median fluorescence intensity units.

Supplementary Figure 8 Correlation of IL-6 with antibodies levels to SARS-CoV-2 proteins in the severe plus critical group. Spearman correlation and non-linear regression (line) were performed between anti-nucleoprotein (NP) IgG, IgA and IgM **(A)**, anti-Spike IgG and IgA **(B)**, and anti-RBD IgG and IgA **(C)** antibodies and IL-6 MFI level values (log scale), in the several plus critical group. Concentration of IL-6 was detected by CBA (28). The presence of antibodies in the serum of patients was measured by enzyme-linked immunosorbent assay (ELISA). Spearman R and p values indicated for each isotype. MFI indicates average of median fluorescence intensity units.

Supplementary Figure 9 Correlation of IL-2 with antibodies levels to SARS-CoV-2 proteins. Spearman correlation and non-linear regression (line) were performed between anti-nucleoprotein (NP) IgG, IgA and IgM **(A)**, anti-Spike IgG and IgA **(B)**, and anti-RBD IgG and IgA **(C)** antibodies and IL-2 MFI level values, in the several plus critical group. Concentration of IL-2 was detected by CBA (28). The presence of antibodies in the serum of patients was measured by enzyme-linked immunosorbent assay (ELISA). Spearman R and p values indicated for each isotype. MFI indicates average of median fluorescence intensity units.

Supplementary Figure 10 Correlation of IL-2 with antibodies levels to SARS-CoV-2 proteins in the fatal group. Spearman correlation and non-linear regression (line) were performed between anti-Spike IgG and IgA **(A)**, anti-nucleoprotein (NP) IgG, IgA and IgM anti-Spike IgG and IgA **(B)**, and anti-RBD IgG and IgA **(C)** with IL-2 of the MFI level values in the fatal group. Concentrations of the IL-2 was detected by CBA (28). The presence of antibodies in the serum of patients was measured by enzyme-linked immunosorbent assay (ELISA). Spearman R and p values indicated for each isotype. MFI indicates average of median fluorescence intensity units.

Supplementary Figure 11 Frequency antibody response to SARS-CoV-2 proteins distinguish survival and fatal patients with COVID-19. Frequency of antibody response in COVID-19 according to fatal (n=36) and survival (n=41). Detection of anti-nucleoprotein (NP), IgG **(A)**, IgA **(B)** and IgM **(C)**, anti-spike IgG **(D)** and IgA **(E)**, anti-RBD IgG **(F)** and IgA **(G)** protein antibodies were measured by enzyme-linked immunosorbent assay (ELISA) as described in Methods. Fisher’s exact test was used for comparison of antibody response between groups; horizontal bar indicates the percentage of frequency with antibodies levels. The line in the middle of box indicates median values. Mann-Whitney test was used for comparison of antibody response between groups. Statistical significances are shown as *p< 0.05, **p < 0.01.

Supplementary Figure 12 Antibody response to SARS-CoV-2 proteins distinguish survival and fatal patients with COVID-19 were analyzed between >30 and ≤60 days after the onset of symptoms. The presence of anti-nucleoprotein (NP) IgG **(A)**, IgM **(B)** and IgA **(C)**, anti-Spike IgG **(D)** and IgA **(E)** and anti-RBD IgG **(F)** and IgA **(G)** protein antibodies were measured by enzyme-linked immunosorbent assay (ELISA) as described in Methods. Results are expressed as sample/cutoff OD (S/CO) as described in Methods. Boxes represent the 25th to 75th percentiles. Each dot represents a single individual, with distribution in maximum and minimum values. The line in the middle of the box indicates median values. Mann-Whitney test was used for comparison of antibody response between groups. Statistical significances are shown as *p< 0.05, **p < 0.01.

Supplementary Figure 13 Antibody response to SARS-CoV-2 proteins distinguish survival and fatal patients with COVID-19 according to the age. Serum of patients with age 40-59 years was analyzed for the presence of anti-NP IgG, IgA and IgM, anti-Spike, and anti-RBD IgG and IgA **(A)**. Serum of patients with age ≥60 years was analyzed for the presence of IgG, IgA and IgM antibodies to NP, IgG and IgA antibodies to Spike and to RBD **(B)**. Antibodies in the serum of patients who survived and those who died was measured by enzyme-linked immunosorbent assay (ELISA). Results are expressed as sample/cutoff OD (S/CO) as described in Methods. Boxes represent the 25th to 75th percentiles. Each dot represents a single individual, with distribution in maximum and minimum values. The line in the middle of box indicates median values. Mann-Whitney test was used for comparison of antibody response between groups. Statistical significances are shown as *p< 0.05, **p < 0.01.

Supplementary Figure 14 Correlations among age, days PSO, blood count, biochemical parameters, and antibodies to SARS-CoV-2 proteins in total cohort of COVID-19 patients. Correlation matrix plot of all variables to identify potential inter-variables correlations. The color represents the direction of the correlation. Spearman R values are shown from red (−1.0) to blue (1.0). Only boxes showing a significant correlation and r > 0.5 have been highlighted. Blank fields indicate lack of signal. Abbreviations: Hb., Hemoglobin; Neutro., Neutrophil; Lympho. Lymphocyte; Mono., Monocyte; PSO., post-symptoms onset; TAP., partial thromboplastin time; TTPA., activated partial thromboplastin time; CRP., C-reactive protein; ALT., Alanine aminotransferase; AST., Aspartate aminotransferase; nAb. Neutralizing antibody. Statistical significances are shown as *p< 0.05, **p < 0.01, ***p < 0.001.

Supplementary Figure 15 Correlations among lymphocyte, monocytes, neutrophil, platelet, erythrocyte, hemoglobin, D-dimer, PCR, Ferritin, and antibodies to SARS-CoV-2 proteins, with mild **(A)**, moderate **(B)**, severe **(C)**, critical **(D)** COVID-19 patients. Correlation matrix plot of all variables to identify potential inter-variables correlations. Spearman R values are shown from red (−1.0) to blue (1.0). Only boxes showing a significant correlation and r > 0.5 have been highlighted. Blank fields indicate lack of signal. Abbreviations: Hb., Hemoglobin; Neutro., Neutrophil; Lympho. Lymphocyte; Mono., Monocyte; PSO., post-symptoms onset; TAP., partial thromboplastin time; TTPA., activated partial thromboplastin time; CRP., C-reactive protein; ALT., Alanine aminotransferase; AST., Aspartate aminotransferase; nAb. Neutralizing antibody. Statistical significances are shown as *p< 0.05, **p < 0.01, ***p < 0.001.
