# Supplementary figures and images for: Distinct anti-NP, anti-RBD and anti-Spike antibody profiles discriminate death from survival in COVID-19
Source: Front Immunol. 2023 Oct 9;14:1206979. doi: 10.3389/fimmu.2023.1206979 (PMC10591157; doi:10.3389/fimmu.2023.1206979)

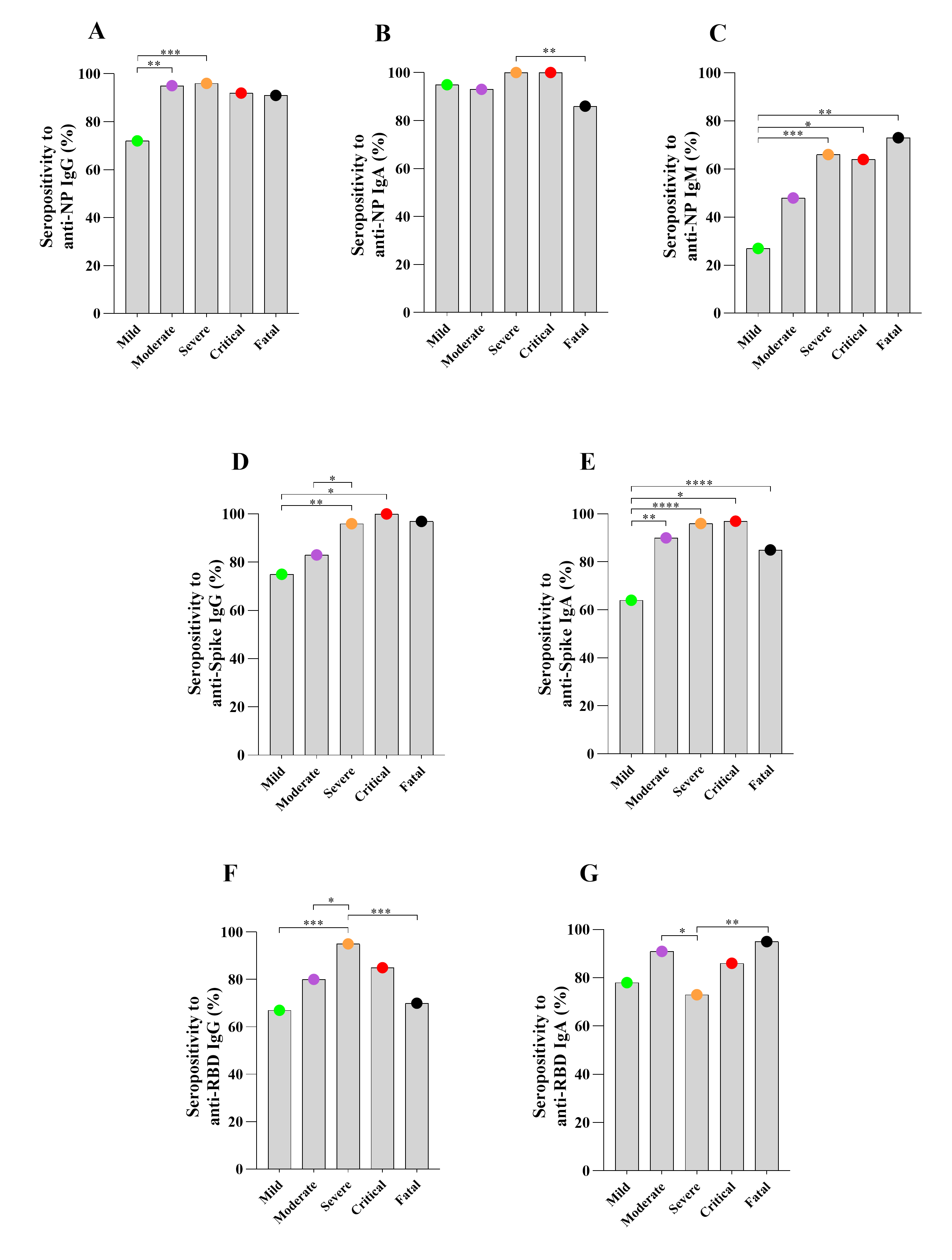

Supplement: Supplementary Figure 1 — Seropositivity to SARS-CoV-2 proteins in COVID-19 patients according to disease severity. Patients with different clinical status were analyzed in the first 30 days post-symptoms onset (mild, n = 37; moderate, n = 43; severe, n = 63; critical n = 13, fatal, n = 36). Detection of anti-nucleoprotein (NP) IgG (A), IgA (B) and IgM (C), anti-Spike IgG (D) and IgA (E) protein and anti-RBD IgG (F) and IgA (G) antibodies were measured by enzyme-linked immunosorbent assay (ELISA) as described in Methods. Results are expressed as frequency of positive response considered with index > 1.2 calculated as the ratio of sample/cutoff OD (S/CO). Fisher’s exact test was used for comparison of antibody response between groups. *p< 0.05, **p < 0.01, ***p < 0.001 and ****p <0.0001. [file Image_1.tif]

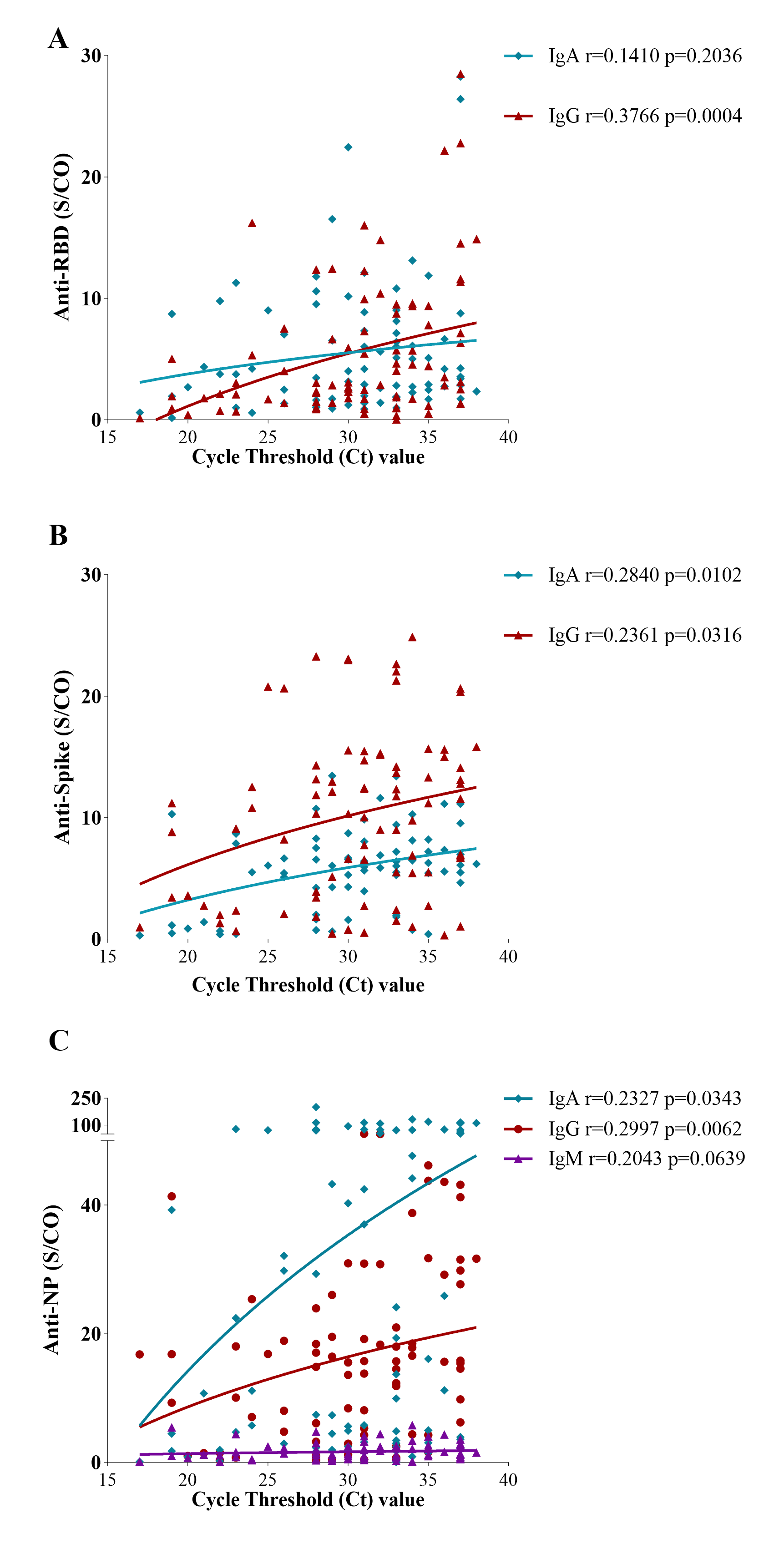

Supplement: Supplementary Figure 2 — Ct values reflect and antibodies levels to SARS-CoV-2 proteins. Spearman correlation and non-linear regression (line) were performed between anti-RBD IgG and IgA (A), anti-Spike IgG and IgA (B), and anti-nucleoprotein (NP) IgG, IgA and IgM (C), and with Ct value, performed by RT-PCR. The presence of antibodies in the patients’ (each point) serum was measured by enzyme-linked immunosorbent assay (ELISA). Spearman R and p values are included each one graphic. [file Image_2.tif]

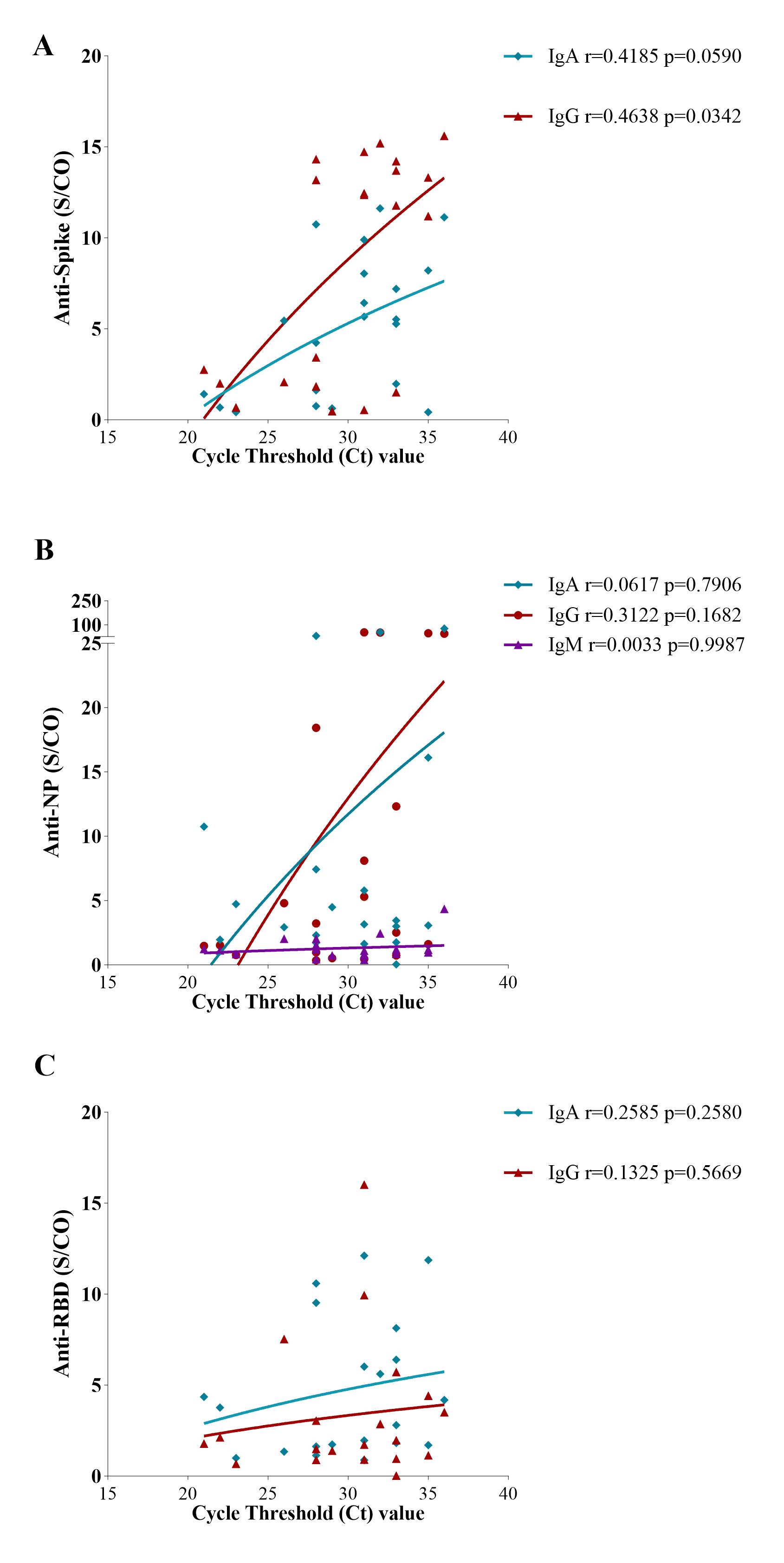

Supplement: Supplementary Figure 3 — Ct values reflect and antibodies levels to SARS-CoV-2 proteins in the mild group. Spearman correlation and non-linear regression (line) were performed between anti-Spike IgG and IgA (A), anti-nucleoprotein (NP) IgG, IgA and IgM (B), and anti-RBD IgG and IgA (C) with Ct value, performed by RT-PCR, in the mild group. The presence of antibodies in the patients’ (each point) serum was measured by enzyme-linked immunosorbent assay (ELISA). Spearman R and p values are included each one graphic. [file Image_3.tif]

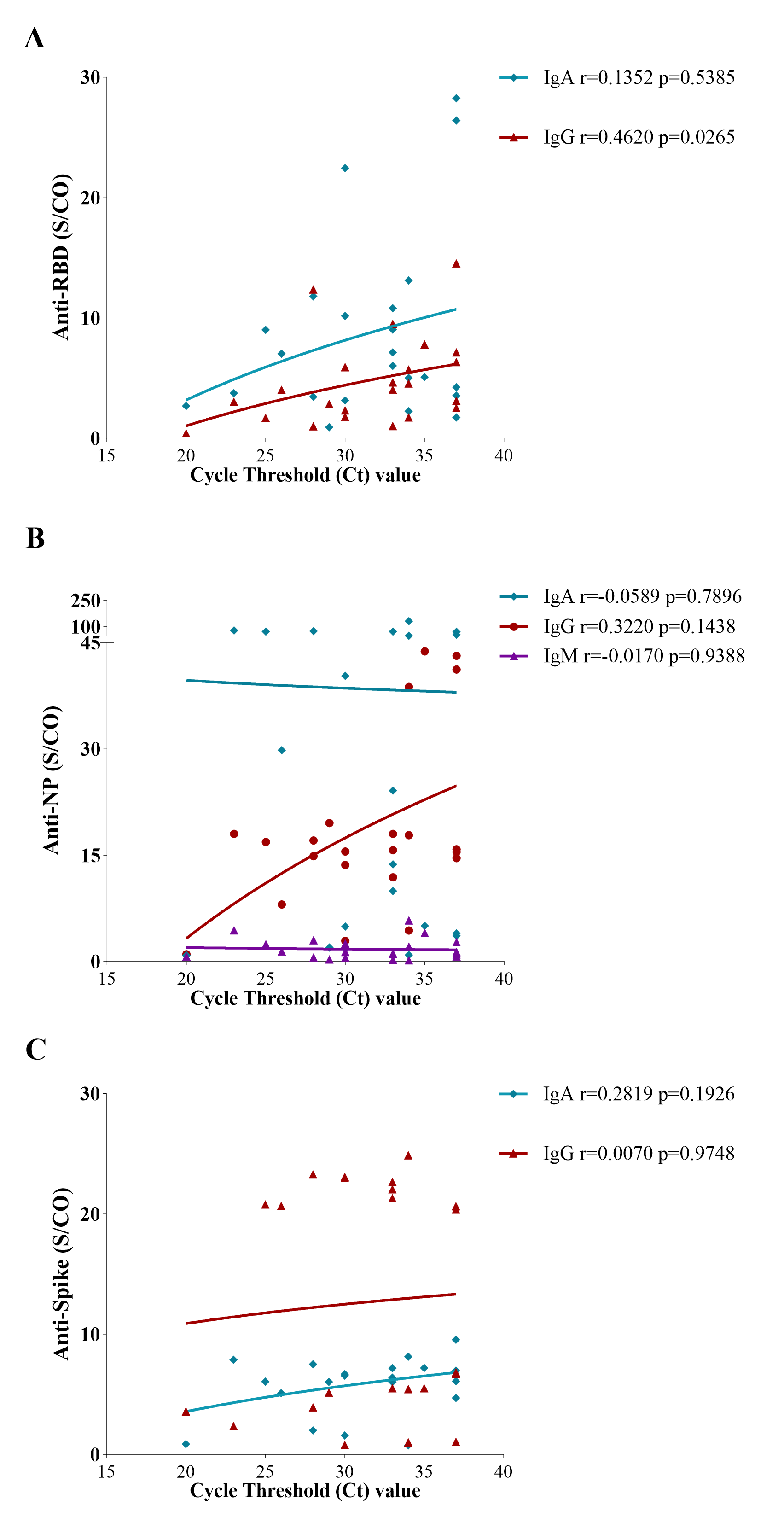

Supplement: Supplementary Figure 4 — Ct values reflect and antibodies levels to SARS-CoV-2 proteins in the moderate group. Spearman correlation and non-linear regression (line) were performed between anti-RBD IgG and IgA (A), anti-nucleoprotein (NP) IgG, IgA and IgM (B), and anti-Spike IgG and IgA (C) with Ct value, performed by RT-PCR, in the moderate group. The presence of antibodies in the patients’ (each point) serum was measured by enzyme-linked immunosorbent assay (ELISA). Spearman R and p values are included each one graphic. [file Image_4.tif]

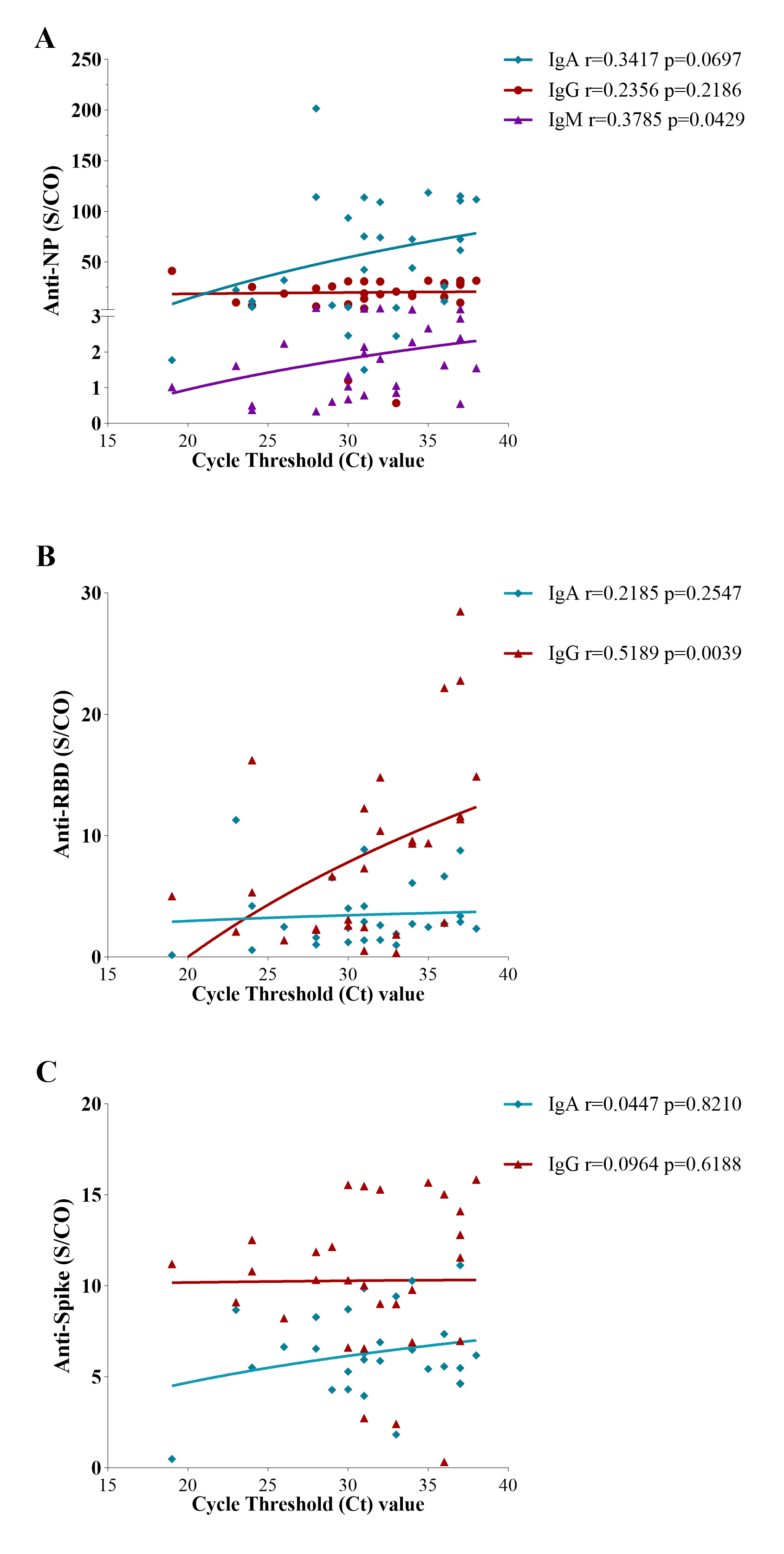

Supplement: Supplementary Figure 5 — Ct values reflect and antibodies levels to SARS-CoV-2 proteins in the severe plus critical group. Spearman correlation and non-linear regression (line) were performed between anti-nucleoprotein (NP) IgG, IgA and IgM (A), anti-RBD IgG and IgA (B), and anti-Spike IgG and IgA (C) with Ct value, performed by RT-PCR, in the severe plus critical group. The presence of antibodies in the patients’ (each point) serum was measured by enzyme-linked immunosorbent assay (ELISA). Spearman R and p values are included each one graphic. [file Image_5.tif]

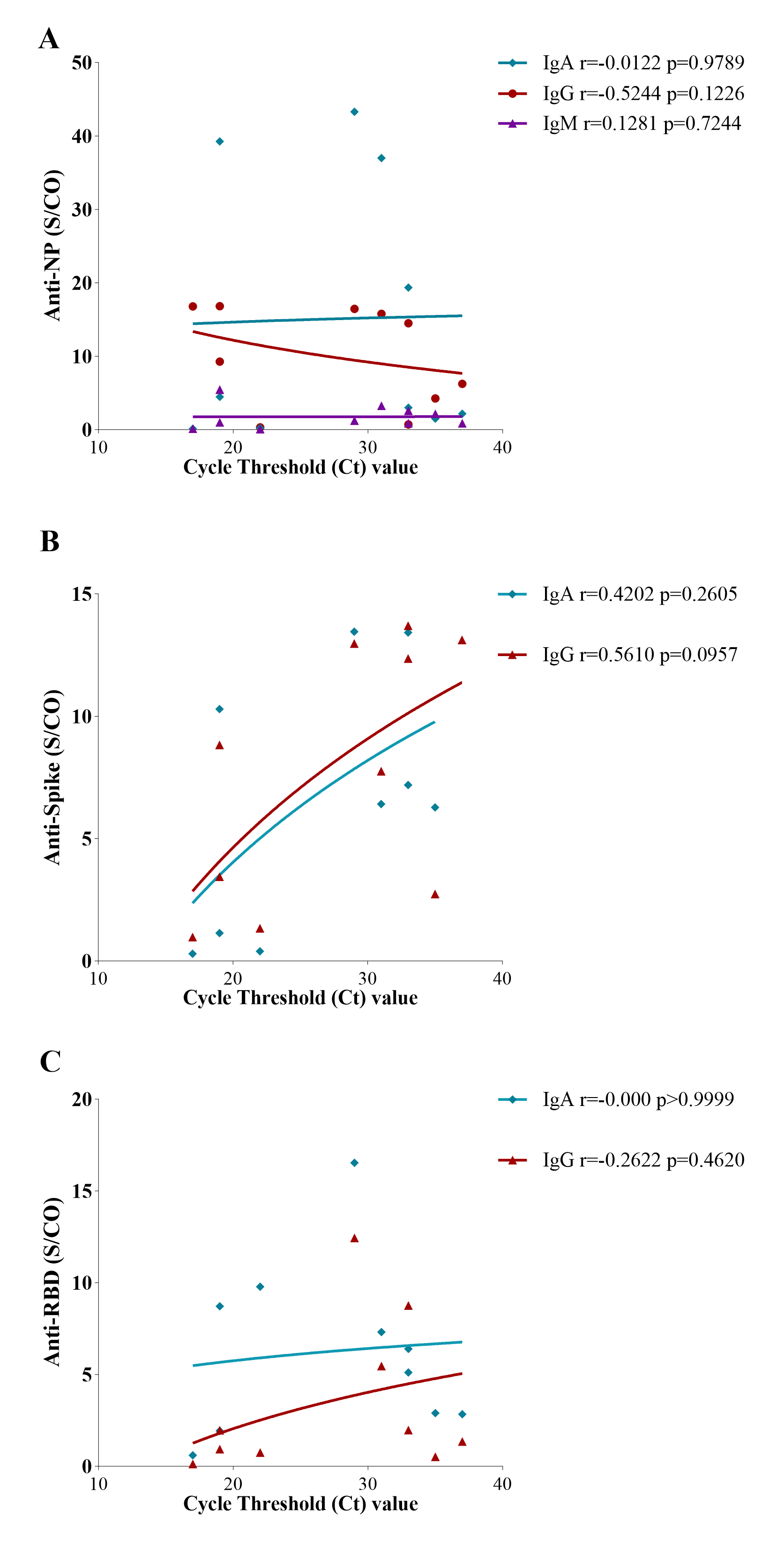

Supplement: Supplementary Figure 6 — Ct values reflect and antibodies levels to SARS-CoV-2 proteins in the fatal group. Spearman correlation and non-linear regression (line) were performed between anti-nucleoprotein (NP) IgG, IgA and IgM (A), anti-Spike IgG and IgA (B), and anti-RBD IgG and IgA (C) with Ct value, performed by RT-PCR, in the fatal group. The presence of antibodies in the patients’ (each point) serum was measured by enzyme-linked immunosorbent assay (ELISA). Spearman R and p values are indicated for each isotype. [file Image_6.tif]

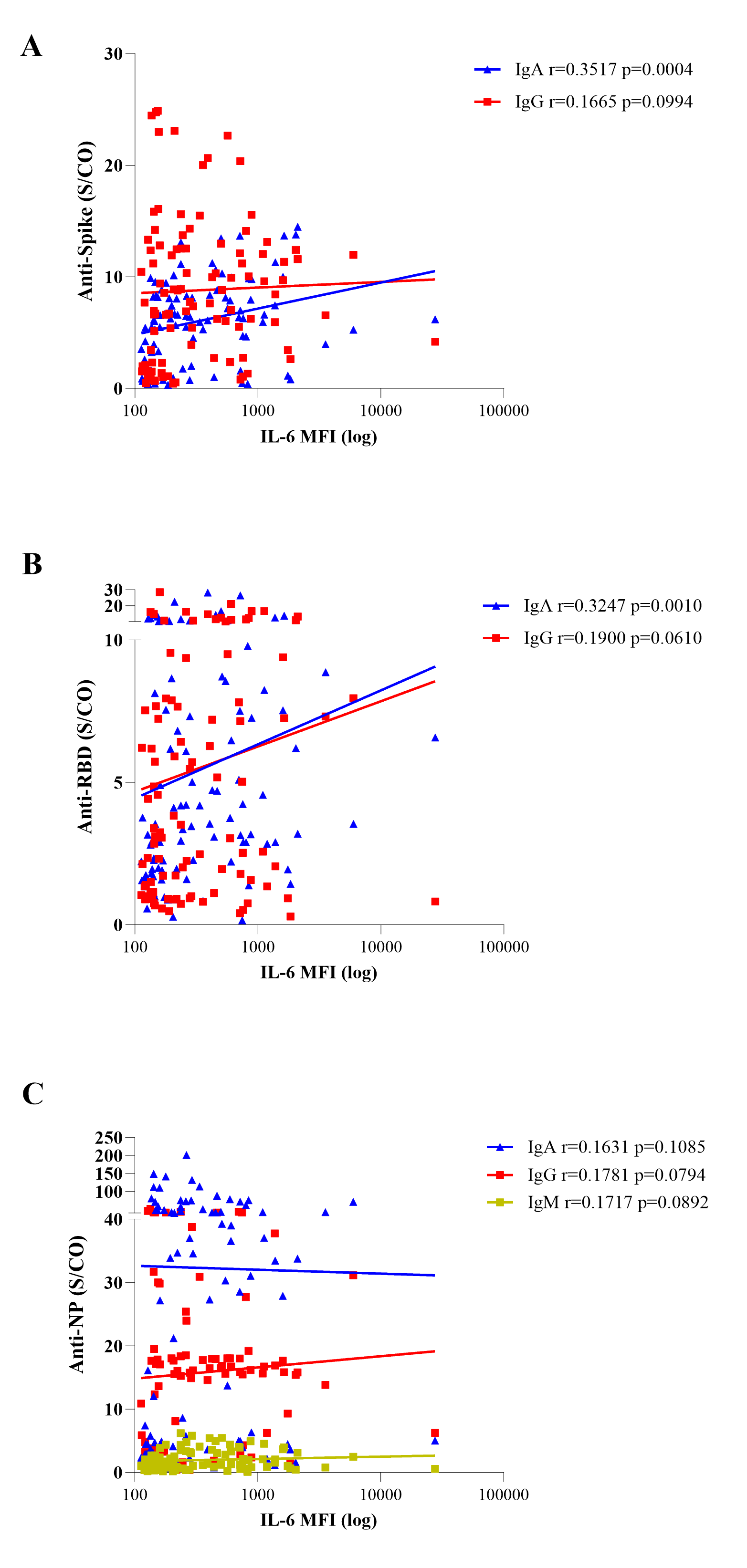

Supplement: Supplementary Figure 7 — Correlation of IL-6 with antibodies levels to SARS-CoV-2 proteins. Spearman correlation and non-linear regression (line) were performed between anti-Spike IgG and IgA (A), anti-RBD IgG and IgA (B), and anti-nucleoprotein (NP) IgG, IgA and IgM (C) antibodies and IL-6 MFI level values (log scale), in the several plus critical group. Concentration of IL-6 was detected by CBA (28). The presence of antibodies in the serum of patients was measured by enzyme-linked immunosorbent assay (ELISA). Spearman R and p values indicated for each isotype. MFI indicates average of median fluorescence intensity units. [file Image_7.tif]

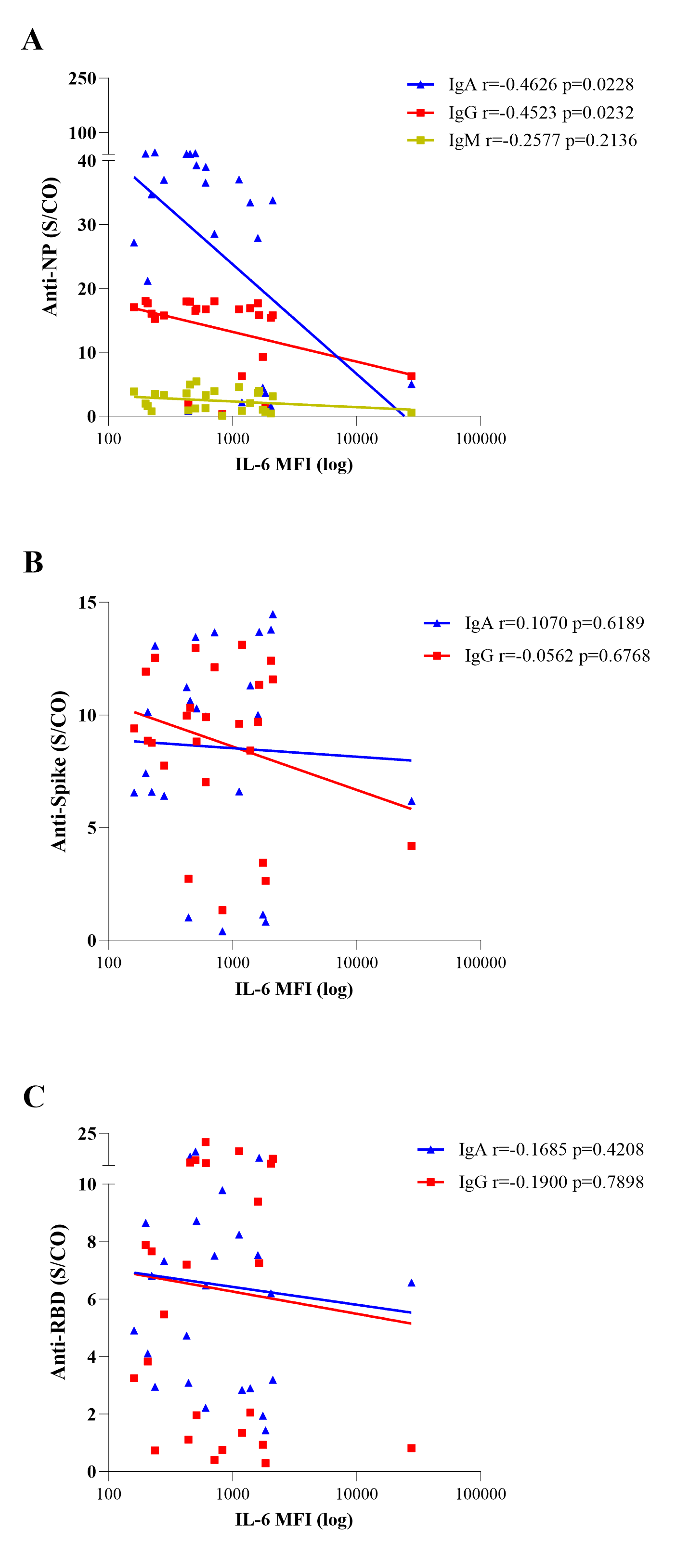

Supplement: Supplementary Figure 8 — Correlation of IL-6 with antibodies levels to SARS-CoV-2 proteins in the severe plus critical group. Spearman correlation and non-linear regression (line) were performed between anti-nucleoprotein (NP) IgG, IgA and IgM (A), anti-Spike IgG and IgA (B), and anti-RBD IgG and IgA (C) antibodies and IL-6 MFI level values (log scale), in the several plus critical group. Concentration of IL-6 was detected by CBA (28). The presence of antibodies in the serum of patients was measured by enzyme-linked immunosorbent assay (ELISA). Spearman R and p values indicated for each isotype. MFI indicates average of median fluorescence intensity units. [file Image_8.tif]

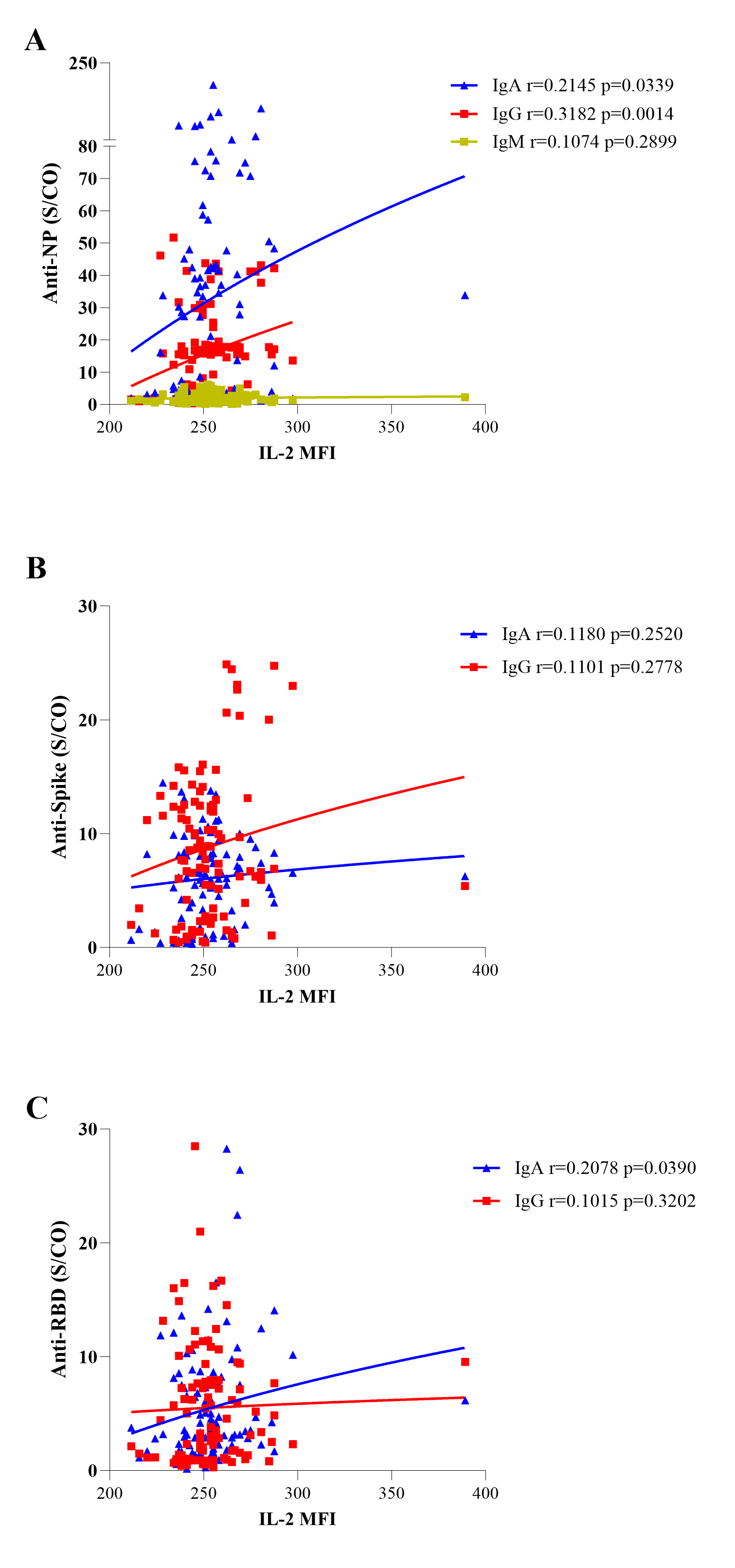

Supplement: Supplementary Figure 9 — Correlation of IL-2 with antibodies levels to SARS-CoV-2 proteins. Spearman correlation and non-linear regression (line) were performed between anti-nucleoprotein (NP) IgG, IgA and IgM (A), anti-Spike IgG and IgA (B), and anti-RBD IgG and IgA (C) antibodies and IL-2 MFI level values, in the several plus critical group. Concentration of IL-2 was detected by CBA (28). The presence of antibodies in the serum of patients was measured by enzyme-linked immunosorbent assay (ELISA). Spearman R and p values indicated for each isotype. MFI indicates average of median fluorescence intensity units. [file Image_9.tif]

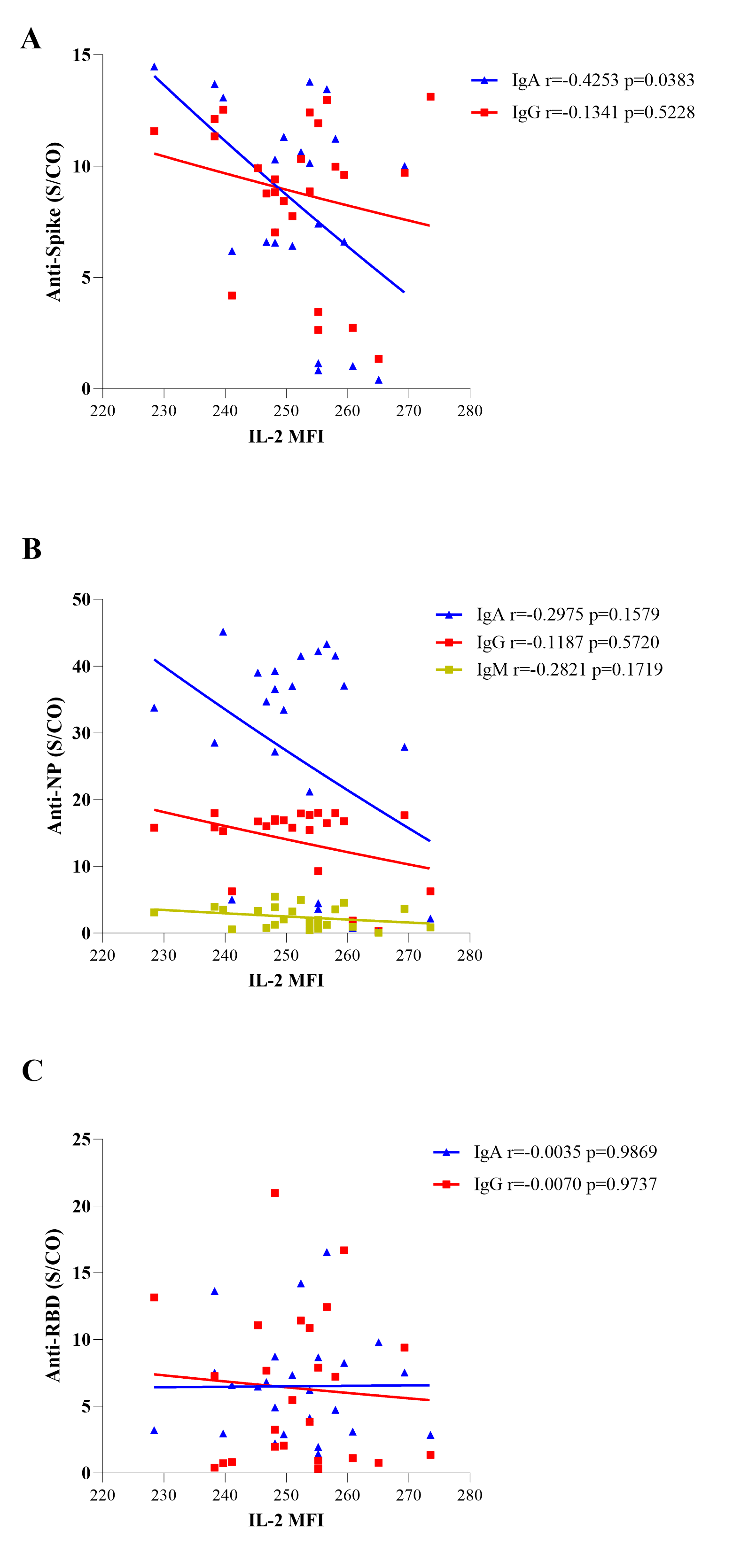

Supplement: Supplementary Figure 10 — Correlation of IL-2 with antibodies levels to SARS-CoV-2 proteins in the fatal group. Spearman correlation and non-linear regression (line) were performed between anti-Spike IgG and IgA (A), anti-nucleoprotein (NP) IgG, IgA and IgM anti-Spike IgG and IgA (B), and anti-RBD IgG and IgA (C) with IL-2 of the MFI level values in the fatal group. Concentrations of the IL-2 was detected by CBA (28). The presence of antibodies in the serum of patients was measured by enzyme-linked immunosorbent assay (ELISA). Spearman R and p values indicated for each isotype. MFI indicates average of median fluorescence intensity units. [file Image_10.tif]

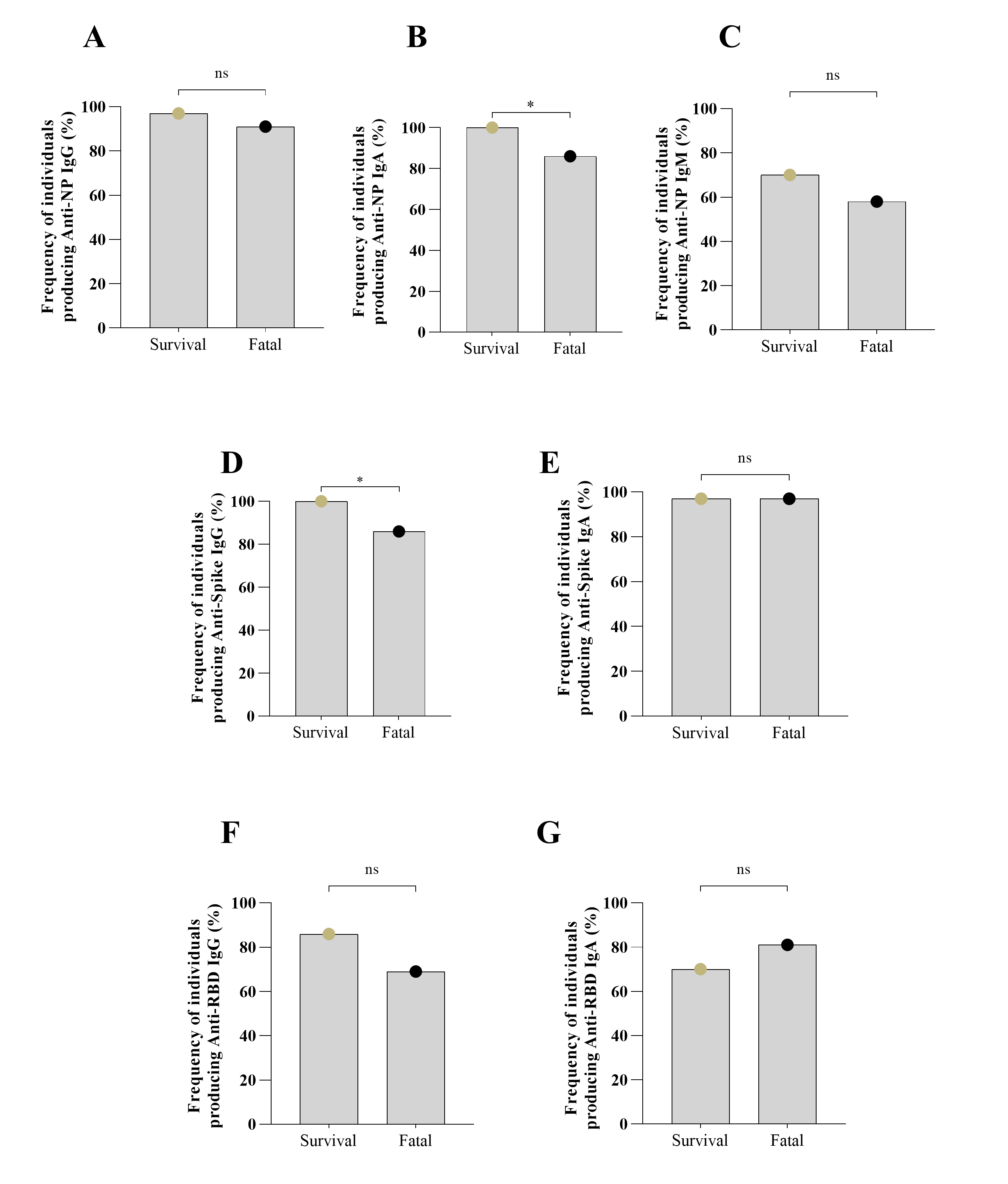

Supplement: Supplementary Figure 11 — Frequency antibody response to SARS-CoV-2 proteins distinguish survival and fatal patients with COVID-19. Frequency of antibody response in COVID-19 according to fatal (n=36) and survival (n=41). Detection of anti-nucleoprotein (NP), IgG (A), IgA (B) and IgM (C), anti-spike IgG (D) and IgA (E), anti-RBD IgG (F) and IgA (G) protein antibodies were measured by enzyme-linked immunosorbent assay (ELISA) as described in Methods. Fisher’s exact test was used for comparison of antibody response between groups; horizontal bar indicates the percentage of frequency with antibodies levels. The line in the middle of box indicates median values. Mann-Whitney test was used for comparison of antibody response between groups. Statistical significances are shown as *p< 0.05, **p < 0.01. [file Image_11.tif]

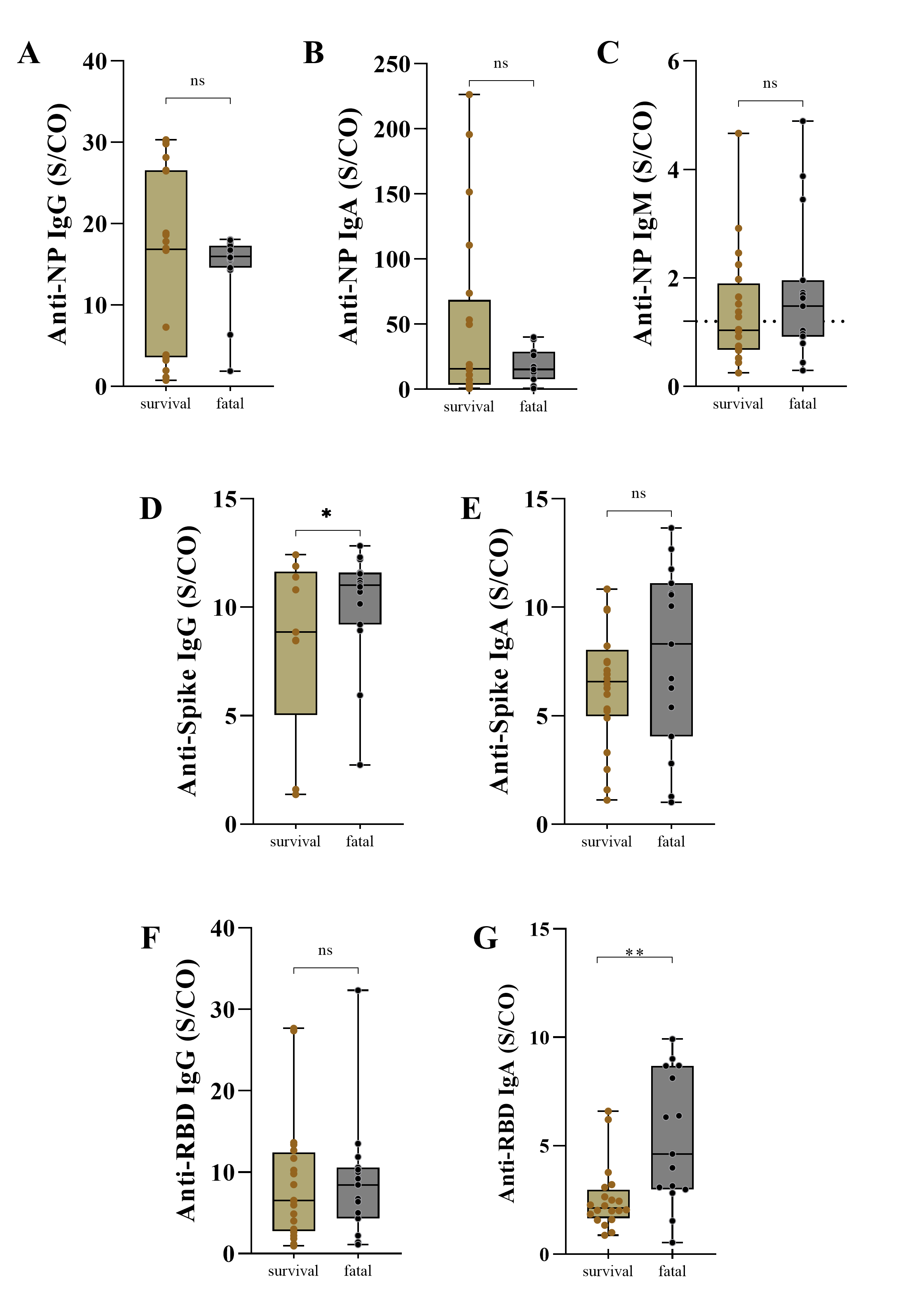

Supplement: Supplementary Figure 12 — Antibody response to SARS-CoV-2 proteins distinguish survival and fatal patients with COVID-19 were analyzed between >30 and ≤60 days after the onset of symptoms. The presence of anti-nucleoprotein (NP) IgG (A), IgM (B) and IgA (C), anti-Spike IgG (D) and IgA (E) and anti-RBD IgG (F) and IgA (G) protein antibodies were measured by enzyme-linked immunosorbent assay (ELISA) as described in Methods. Results are expressed as sample/cutoff OD (S/CO) as described in Methods. Boxes represent the 25th to 75th percentiles. Each dot represents a single individual, with distribution in maximum and minimum values. The line in the middle of the box indicates median values. Mann-Whitney test was used for comparison of antibody response between groups. Statistical significances are shown as *p< 0.05, **p < 0.01. [file Image_12.tif]

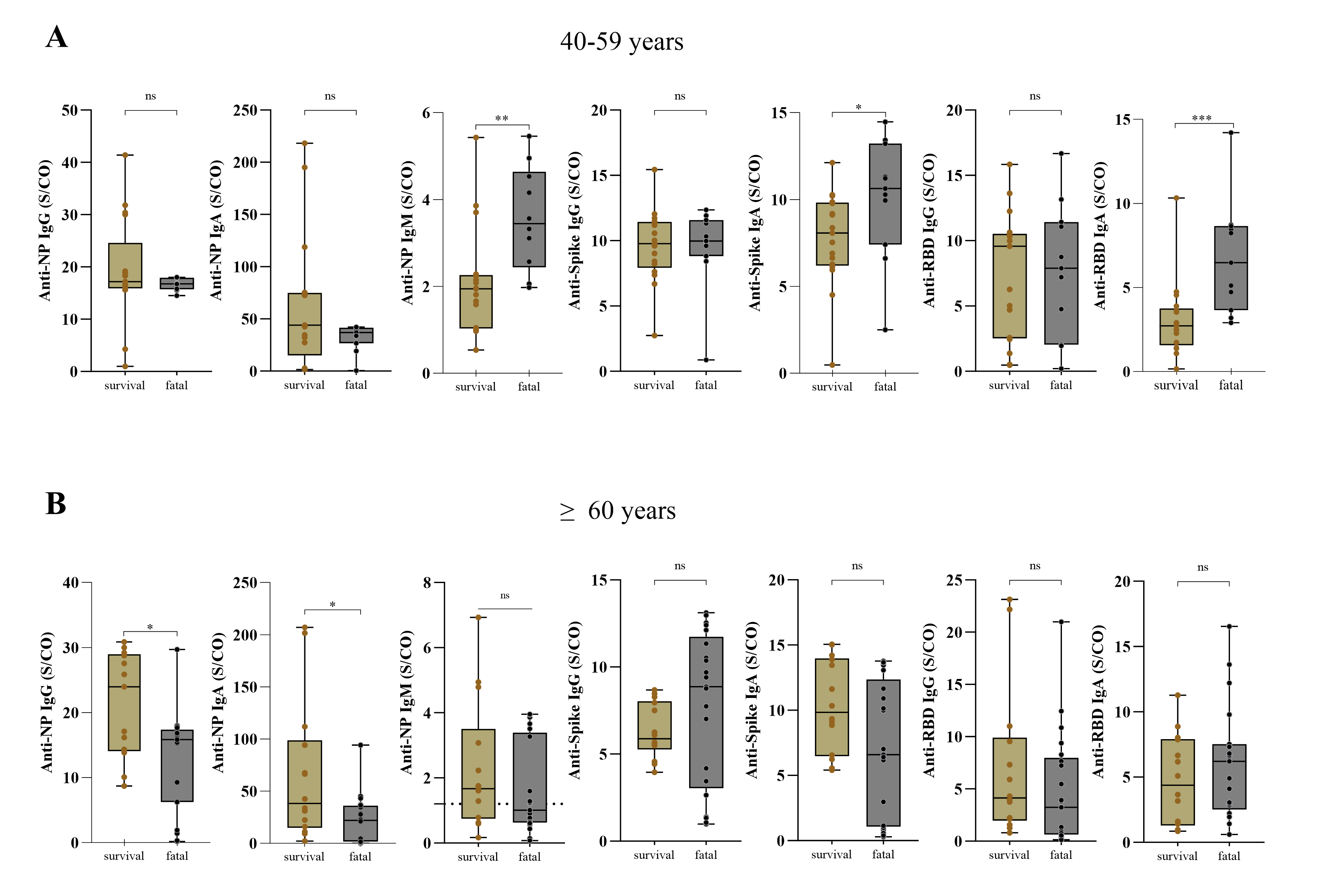

Supplement: Supplementary Figure 13 — Antibody response to SARS-CoV-2 proteins distinguish survival and fatal patients with COVID-19 according to the age. Serum of patients with age 40-59 years was analyzed for the presence of anti-NP IgG, IgA and IgM, anti-Spike, and anti-RBD IgG and IgA (A). Serum of patients with age ≥60 years was analyzed for the presence of IgG, IgA and IgM antibodies to NP, IgG and IgA antibodies to Spike and to RBD (B). Antibodies in the serum of patients who survived and those who died was measured by enzyme-linked immunosorbent assay (ELISA). Results are expressed as sample/cutoff OD (S/CO) as described in Methods. Boxes represent the 25th to 75th percentiles. Each dot represents a single individual, with distribution in maximum and minimum values. The line in the middle of box indicates median values. Mann-Whitney test was used for comparison of antibody response between groups. Statistical significances are shown as *p< 0.05, **p < 0.01. [file Image_13.tif]

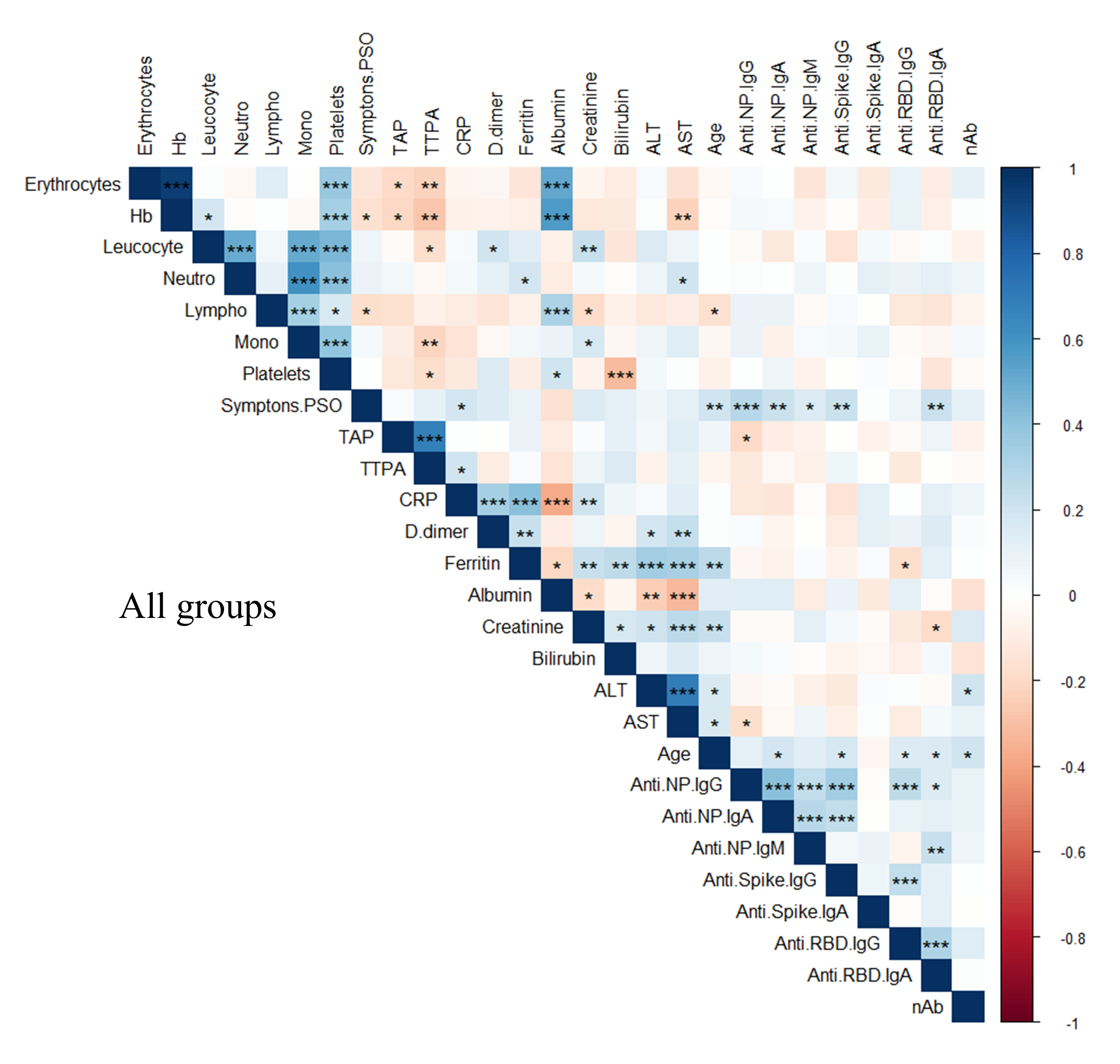

Supplement: Supplementary Figure 14 — Correlations among age, days PSO, blood count, biochemical parameters, and antibodies to SARS-CoV-2 proteins in total cohort of COVID-19 patients. Correlation matrix plot of all variables to identify potential inter-variables correlations. The color represents the direction of the correlation. Spearman R values are shown from red (−1.0) to blue (1.0). Only boxes showing a significant correlation and r > 0.5 have been highlighted. Blank fields indicate lack of signal. Abbreviations: Hb., Hemoglobin; Neutro., Neutrophil; Lympho. Lymphocyte; Mono., Monocyte; PSO., post-symptoms onset; TAP., partial thromboplastin time; TTPA., activated partial thromboplastin time; CRP., C-reactive protein; ALT., Alanine aminotransferase; AST., Aspartate aminotransferase; nAb. Neutralizing antibody. Statistical significances are shown as *p< 0.05, **p < 0.01, ***p < 0.001. [file Image_14.tif]

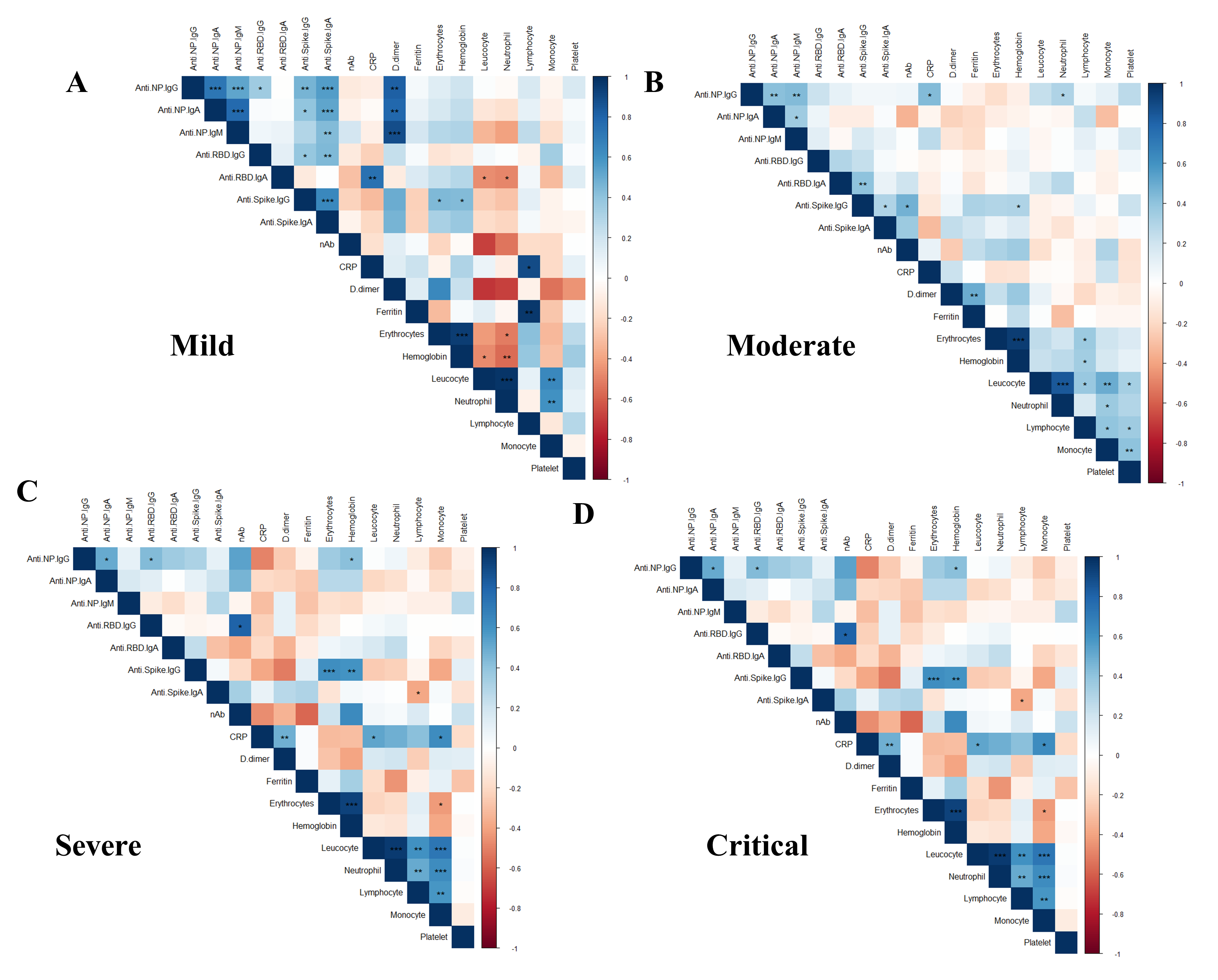

Supplement: Supplementary Figure 15 — Correlations among lymphocyte, monocytes, neutrophil, platelet, erythrocyte, hemoglobin, D-dimer, PCR, Ferritin, and antibodies to SARS-CoV-2 proteins, with mild (A), moderate (B), severe (C), critical (D) COVID-19 patients. Correlation matrix plot of all variables to identify potential inter-variables correlations. Spearman R values are shown from red (−1.0) to blue (1.0). Only boxes showing a significant correlation and r > 0.5 have been highlighted. Blank fields indicate lack of signal. Abbreviations: Hb., Hemoglobin; Neutro., Neutrophil; Lympho. Lymphocyte; Mono., Monocyte; PSO., post-symptoms onset; TAP., partial thromboplastin time; TTPA., activated partial thromboplastin time; CRP., C-reactive protein; ALT., Alanine aminotransferase; AST., Aspartate aminotransferase; nAb. Neutralizing antibody. Statistical significances are shown as *p< 0.05, **p < 0.01, ***p < 0.001. [file Image_15.tif]
